# METTL16 participates in haemoglobin H disease through m^6^A modification

**DOI:** 10.1371/journal.pone.0306043

**Published:** 2024-08-01

**Authors:** Yuping Liao, Feng Zhang, Fang Yang, Shijin Huang, Sha Su, Xuemei Tan, Linlin Zhong, Lingjie Deng, Lihong Pang

**Affiliations:** 1 Department of Prenatal Diagnosis, The First Affiliated Hospital of Guangxi Medical University, Nanning, Guangxi, China; 2 Center of Reproductive Medicine, Seven Affiliated Hospital of Guangxi Medical University (Wuzhou Gongren Hospital), Wuzhou, Guangxi, China; 3 Department of Obstetrics and Gynecology, The First Affiliated Hospital of Guangxi Medical University, Nanning, Guangxi, China; 4 Guangxi Key Laboratory of Thalassemia Research, Nanning, Guangxi, China; 5 NHC Key Laboratory of Thalassemia Medicine (Guangxi Medical University), Nanning, Guangxi, China; 6 Key Laboratory of Early Prevention and Treatment for Regional High Frequency Tumor (Guangxi Medical University), Ministry of Education, Nanning, Guangxi, China; 7 Guangxi Key Laboratory of Early Prevention and Treatment for Regional High Frequency Tumor, Nanning, Guangxi, China; North Carolina State University, UNITED STATES

## Abstract

**Background:**

Haemoglobin H (HbH) disease is caused by a disorder of α-globin synthesis, and it results in a wide range of clinical symptoms. M^6^A methylation modification may be one of the mechanisms of heterogeneity. Therefore, this article explored the role of methyltransferase like 16 (METTL16) in HbH disease.

**Method:**

The results of epigenetic transcriptome microarray were analysed and verified through bioinformatic methods and qRT-PCR, respectively. The overexpression or knock down of METTL16 in K562 cells was examined to determine its role in reactive oxygen species (ROS), cell cycle processes or iron overload. YTH domain family protein 3 (YTHDF3) was knocked down in K562 cells and K562 cells overexpressing METTL16 via siRNA to investigate its function. In addition, haemoglobin expression was detected through benzidine staining. qRT-PCR, WB, methylated RNA Immunoprecipitation (MeRIP) and (RNA Immunoprecipitation) RIP experiments were conducted to explore the mechanism of intermolecular interaction.

**Results:**

METTL16, YTHDF3 and solute carrier family 5 member 3 (SLC5A3) mRNA and the methylation level of SLC5A3 mRNA were downregulated in HbH patients. Insulin-like growth factor 2 mRNA-binding protein 3 (IGF2BP3) mRNA expression was negatively correlated with HGB content among patients with HbH-CS disease. Overexpression of METTL16 increased ROS and intracellular iron contents in K562 cells, changed the K562 cell cycle, reduced hemin-induced haemoglobin synthesis, increased the expressions of SLC5A3 and HBG and increased SLC5A3 mRNA methylation levels. Knockdown of METTL16 reduced ROS and intracellular iron contents in K562 cells. Hemin treatment of K562 cells for more than 14 days reduced the protein expressions of METTL16 and SLC5A3 and SLC5A3 mRNA methylation levels. Knockdown of YTHDF3 rescued the intracellular iron content changes induced by the overexpression of METTL16. The RIP experiment revealed that SLC5A3 mRNA can be enriched by METTL16 antibody.

**Conclusion:**

METTL16 may affect the expression of SLC5A3 by changing its m^6^A modification level and regulating ROS synthesis, intracellular iron and cycle of red blood cells. Moreover, METTL16 possibly affects the expression of haemoglobin through IGF2BP3, which regulates the clinical phenotype of HbH disease.

## Introduction

Thalassemia is an autosomal recessive hemolytic disease caused by defective globin synthesis. It is stratified into two types: α- thalassemia and β- thalassemia. Currently, thalassemia is the most common single-gene disease and has the largest population worldwide. Approximately 5% of the population worldwide carries an α-thalassemia variant [1]. HbH disease is also referred to as intermediate type α-thalassemia. This condition generally consists of three α-globin gene defects, and α symptoms appear because of reduced rates of peptide chain synthesis. Some individuals who are homozygous and double heterozygous for non-deletional mutations may also show HbH-like symptoms (such as α CS and α QS). Due to the lack of α peptide chain, excessive γ peptide chains form tetramers in the fetal period, namely Hb Bart’s, and postnatal excessive β peptide chain form tetramers, namely Hemoglobin H (HbH). The aggregation of HbH inclusion bodies in the red blood cell membrane reduces the shaping ability of the red blood cells, and the red blood cells are easy to break, resulting in hemolysis and anemia. [2]. HbH disease is divided into deletion and nondeletion types based on its genotype. Furthermore, it is generally observed that the clinical manifestations of nondeletion-type HbH disease are more severe than deletion-type [3]. The HbH-CS disease is one of the most severe clinical symptoms in HbH disease. It has a poor prognosis and can manifest as moderate to severe anemia, splenomegaly, jaundice, special facial features, and so on. It may also have various complications, including hemolytic crisis, bone deformity, gallstone, lower limb ulcer, thrombotic disease, and so on [4, 5]. In addition, it seriously affects human health and can lead to disability and/or even death [6]. Simultaneously, clinical observation also found that the clinical manifestations of patients with HbH disease, including HbH-CS disease, also have evident heterogeneity. Some patients have only mild anemia, normal growth and development, no hepatosplenomegaly, and no evident complications; meanwhile, some showed moderate to severe anemia, even transfusion-dependent thalassemia, which affected their growth and development and caused various complications. However, this clinical heterogeneity cannot be explained by pathogenic genes alone, which brings uncertainty to prenatal diagnosis and consultation, thereby resulting in a dilemma when parents of HbH fetuses make choices in clinical prenatal diagnosis. The reason is that in addition to genotype, some regulatory factors and mechanisms affect the phenotype of HbH disease, which may be realized through the mechanism of epigenetic regulation. Prenatal diagnosis of thalassemia generally involves chorionic villus sampling at 11–14 weeks of pregnancy. However, during this period, the foetus is unamenable to routine blood analysis for the assessment of anaemia. Moreover, infants with haemoglobin H (HbH) may not exhibit overt symptoms at birth, with anaemia manifesting gradually after the neonatal period. Therefore, the screening of other phenotypic regulators and prognostic predictors in addition to genotype and the research on epigenetic regulation is urgently needed for scientific and clinical research of HbH disease.

RNA modification (also known as the "epigenetic transcriptome") plays an important role in regulating various gene expressions in recent years. At present, there are over 170 naturally occurring RNA modifications [7] have been found in nature, including m^6^A, m^5^C, and m^1^A. Recent research has focused on the most common RNA modification, N6 methyladenine (m^6^A). It plays a role in post-transcriptional regulation by regulating the splicing, translation, stability, structure, transportation, and degradation of mRNA and its precursors, noncoding RNA, ribosomal RNA, and so on [8–12]. Moreover, it plays a key role in various important biological processes, such as cell growth, stem cell self-renewal and differentiation, tumorigenesis, hematopoiesis, viral replication, inflammation, immune response, adipogenesis, embryonic development, and osteogenesis [13–16]. Methyltransferase 16, N6 methyladenosine is a type of m^6^A "writer". Human methyltransferase 16 (METTL16) contains 562 amino acid residues It also contains a highly conserved N-terminal methyltransferase domain (MTD) and a C-terminal domain composed of two vertebrate conserved regions (VCRs) [17, 18]. Furthermore, it is an RNA-binding protein that is expressed in both the nucleus and cytoplasm [19]. In the METTL family, METTL3 and METTL14 have been studied more and have strong functions. At present, it is believed that in eukaryotes, most m^6^A modifications are completed by the METTL3/METTL14 complex [20], compared with METTL16 which has relatively few studies. Recently, METTL16 also regulates gene expression and has both methyltransferase activity-dependent and -independent functions: In the nucleus, METTL16 promotes m^6^A modification of downstream RNA as a "writer" of m^6^A; in the cytoplasm, METTL16 promotes translation in a way independent of m^6^A [21]. In addition, its confirmed targets include lncRNA MALAT1, lncRNA XIST, snRNA U6, and mRNA MAT2A [22]. It also participates in biological processes such as tumorigenesis [23] DNA damage [24], cell apoptosis, and oxidative stress [25].

Sornjai et al. [26] found that the change of ribosomal RNA methylation mode may lead to translation defects in thalassemia patients. Our research group found that m^6^A plays a significant role in thalassemia. M^6^A writers including METTL16, WTAP (WT1 associated protein), CBLL1 (Cbl proto-oncogene like 1), RBM15B (RNA binding motif protein 15B), and ZC3H13 (zinc finger CCCH-type containing 13) and reader proteins including IGF2BP2 (insulin like growth factor 2 mRNA binding protein 2) and YTHDF3 (YTH N6-methyladenosine RNA binding protein F3) are differentially expressed between HbH-CS patients and normal people [27], however, the mechanistic study of the role of METTL16 in HbH disease and the correlation analysis between the expression level of m^6^A related enzymes and the severity of HbH-CS anemia have not been reported in the investigated literature. In this study, we analyzed the link between epigenetics and the HbH-CS phenotype using the human m^6^A-mRNA and lncRNA epitranscriptomic microarray data, isolated nucleated red blood cells (NRBCs), and reticulocytes from the peripheral blood of HbH thalassemia as well as healthy controls to validate the chip results in clinical samples and further clarify the function and mechanism of METTL16 in vitro using K562 cells. K562 cells can express embryonic and fetal hemoglobin when cultured under normal conditions. The protein level in the β gene cluster mainly expresses HBG and HBE. The protein level in the α gene cluster mainly expresses more HBZ and less HBA. HBZ is upregulated in the peripheral blood of some α -thalassemia carriers [28]. We used K562 cell line as the cell model of this study to provide new ideas for prenatal counseling and treatment of HbH disease.

## Materials and methods

### Study participants

This study was approved by the Declaration of Helsinki and the Medical Ethics Committee of the First Affiliated Hospital of Guangxi Medical University.

All participants in this study were recruited from the First Affiliated Hospital of Guangxi Medical University from May 1, 2020 to March 30, 2023. All subjects signed an informed consent form before the trial began. All HbH patients confirmed their diagnosis with blood routine, hemoglobin electrophoresis, and DNA analysis. Meanwhile, HbH patients did not receive any blood transfusion in the past 3 months and without any other blood-related diseases. Healthy controls were considered without any blood-related diseases. The diagnostic standard for severe anemia is Hb<60g/L. Authors had not access to information that could identify individual participants during or after data collection.

Isolation of NRBC and reticulocytes. Peripheral blood mononuclear cells, NRBC, and reticulocytes were separated by density gradient centrifugation using human peripheral blood lymphocyte isolate fluid (TBD, Tianjin, China), reticulocytes and nucleated erythrocytes are about 30–60% pure [29]. Further enrichment was performed using CD71 (CD71 Microbeads human, Miltenyi Biotec GmbH, Germany, 130-046-201) -positive selection with magnetic scaffolds (MiniMACS Starting Kit, Miltenyi Biotec GmbH, Germany) and confirmed by flow cytometry. The flow cytometry results are depicted in S1 Fig in S1 File. Total RNA isolation and qRT-PCR analysis.

Using the extracted total RNA with RNAiso Plus (Takara, 9108), cDNA was synthesized with Prime Script TMRT reagent Kit with GDNA Eraser (Perfect Real-Time; Takara Bio, Shiga, Japan, RR047B). Then, RT-PCR quantitative analysis was performed using the QuantiNova SYBR Green PCR kit (QIAGEN, 208056). Human β-actin was used as a reference control, and the 2^-ΔΔCt^ method to determine its relative expression. Primers are compiled in S1 Table in S1 File.

Human m^6^A-mRNA&lncRNA epitranscriptomic microarray analysis.

Our research group previously tested human m^6^A-mRNA and long non-coding RNA (lncRNA) epitranscriptomic microarray of nucleated erythrocytes and reticulocytes from five healthy volunteers (N) and five HbH-CS thalassemia patients (T). Herein, we performed an additional quantile normalization method of the limma package which was used to normalize the RNA expression level between arrays before probes flag screening. Differences in the m^6^A-methylated RNAs or expressed genes were determined using fold change and statistical significance (*p*-value) thresholds. The preset threshold was FC>1.5, or <1/1.5, *p* < 0.05. Hierarchical clustering was performed using the R software.

Pearson’s correlation analysis was used to determine the correlations between m^6^A mRNA and lncRNA epitranscriptomic microarray methylation level or expression level and hematological parameters HBH, HbF, HbA2, HGB, MCV, MCH, MCHC, and SF. Absolute values |Pearson R|>0.80 and *P-*value < 0.05 were used for further analysis.

### Immunofluorescence

We evenly spread the selected suspension of nucleated red blood cells and reticulocytes on a glass slide, fixed the cells with 4% paraformaldehyde (Absin, abs9179), then we used 0.01% Triton X-100 (Solarbio, T8200) to penetrate the cell membrane and sealed the slide with BSA. Then, we incubated the cells with primary antibodies. Primary antibodies were specific for METTL16 (1:100; Abcam, UK, ab252420), SLC5A3 (1:100; Proteintech, China, 21628-1-AP) and YTHDF3 (1:100; Invitrogen, PA5-97034). Then we incubated the cells with secondary antibodies (goat anti rabbit fluorescent secondary antibody, 1:200; Abmart, M21014). We restained the nucleus and observe it under a fluorescence microscope. We use Image J software to quantitatively analyze the mean fluorescence intensity of images.

### MeRIP-qPCR

Total RNA was extracted from pretreated cells and was immunoprecipitated using magnetic beads pre-coated with 2 ug of anti-m^6^A antibody (Epigentek, P-9018 24) or anti-rabbit IgG (Epigentek, P-9018 24). Then, they were randomly fragmented into sections between 30–200 bps. The RNA samples were then placed in tubes on the magnetic device until the solution was clear. The RNA release was then performed, and they were subsequently purified and recovered for downstream qPCR analysis. We found methylation sites in SLC5A3 on the website http://m6avar.renlab.org/. We then selected position Chr21:34095157 and designed the corresponding primers for qPCR SLC5A3 Forward 5 ′TGCTGTGTTGCTCTGTGTAGT 3 ′, SLC5A3 Reverse 5 ′ GGCCACTATGGCAATGTCTG 3 ′.

### Cell culture and transfection

K562 cell lines (Procell Biotechnology Co., Ltd., Wuhan, China) were cultured on RPMI matrix 1640 (Gibco) consisting of 10% fetal bovine serum (Oricell, Uruguay) and 1% penicillin-streptomycin (Solarbio, Beijing, China). Stable transfection: K562 cells were stably transformed using the methyltransferase like 16 (METTL16) gene-overexpression lentivirus. (pLenti-EF1-EGFP-P2A-Puro-CMV-METTL16-3xFLAG-WPRE), lentivirus control vector, lentiviral-based short-hairpin RNA (shRNA) (pSLenti-U6-shRNA(METTL16)-CMV-Puro-WPRE) and shRNA control vector from OBIO Technology (Shanghai, China). Infected cells were selected with 4 μg/ml puromycin medium for 1 week. Transient transfection: si-YTH domain family protein 3 (YTHDF3) and siCtrl were procured through Tsingke (Beijing), with sequences compiled in S2 Table in S1 File.

### Construction of the cell high-iron culture environment

We cultured cells with iron-containing reagent hemin medium at a final concentration of 40 μM for more than 14 days to mimic the high-iron environment in the blood of patients with HbH disease.

### Western blotting

Total protein was extracted from cells using RIPA buffer (Beyotime, China, P0013B) containing PMSF (Beyotime, China, ST505). Samples were consequently segregated through SDS-PAGE, and transported onto polyvinylidene difluoride (PVDF) membranes, followed by 5% non-fat milk blocking (120 min/RT), with eventual primary and secondary antibody staining before imaging. Primary antibodies were specific for METTL16 (1:1000; Abcam, UK, ab252420), SLC5A3 (1:1000; Proteintech, China, 21628-1-AP), HBG (1:1000; Invitrogen, USA, JM84-10),YTHDF3 (1:1000; Invitrogen, PA5-97034) and antibodies for GAPDH (1:5000; Abmart, China, AB_2737054) served as internal controls. Goat anti-rabbit IgG (H+L) DyLight 800–4 X PEG (1:10000; Invitrogen, USA, SA5-35571) combined with Goat anti-mouse IgG (H+L) antibody DyLight 800–4 X PEG (1:10000; Invitrogen, USA, SA5-35521) was used when near-infrared fluorescent gel imaging system was applied. In addition, HRP-coupled secondary antibodies were used when the chemiluminescence imaging system was applied. ImageJ software and GAPDH were used for densitometric analyses and normalization, respectively.

### Benzidine staining

The stably transformed and controlled K562 cells were seeded into a medium containing hemin (Aladdin,16009-13-5) (final concentration: 40 uM) in 6-well plates. The cells were washed with PBS and stained with benzidine (Aladdin, 92-87-5), and the blue staining of cells was observed at day 5 after incubation.

### Determination of ROS content of cells

DHE (FEIYUBIO, FY17032) with a final concentration of 10 uM was added into cells, incubated at room temperature and away from light for 1 hour. Subsequently, we used a flow cytometer to detect fluorescence intensity and analyzed the data using FlowJo.

### Determination of intracellular iron

The content of iron ions in cells was determined by washing the cells twice with 2 ml of cold PBS, splitting the cells in a shaker for 2 h, adding potassium permanganate mixture to mix well, and incubating at 60°C for 1 h. A total of 30 ul iron ion detector (Intracellular Iron Colorimetric Assay Kit, applygen, E1042) was added, mixed well, and incubated at room temperature for 30 min. In addition, 200 ul was taken to 96 well plates, the absorbance was measured at 550 nm, the standard curve was drawn, and the iron ion concentration was calculated.

### Cell cycle detection

A total of 1×10^6^ logarithmic growth phase cells were collected and washed once with phosphate-buffered saline (PBS). Then, we added 1 ml DNA staining solution (propidium iodide) and 10 μl permeabilisation solution (liankebio, CCS012) to the cells. The cells were incubated at room temperature for 30 min without light and then examined via flow cytometry.

### Cell Counting Kit-8 (CCK-8) assay

Transfected cells were added to 96-well plates (2000 cells/well). CCK-8 reagent (Biosharp, Beijing, China, BS350B) was added at 1, 2, 3 and 4 days after transfection and incubated in a dark incubator for 1.5 h before the optical density was measured at a wavelength of 450 nm using a microplate reader (BioTek, GuangZhou, China).

### Cell apoptosis experiment

A total of 1–10×10^5^ cells were centrifuged, washed with precooled PBS and resuspended in 500 μl 1×Binding Buffer. Exactly 5 μl Annexin V-APC and 10 μl 7-AAD (MULTI SCIENCES, AP105 60-kit) were added to each tube. After gentle vortex mixing, the cells were incubated at room temperature in the dark for 5 min and then examined using flow cytometry.

### RNA immunoprecipitation

Cells were collected and lysed, then incubated at 4°C overnight through magnetic beads conjugated with 5 μg of anti-METTL16 antibody (Abcam, ab252420) and 5 μg of anti-rabbit IgG (MerckMillipore, RIP-12RXN). Proteinase K treatment was followed by the elution of targeted RNA sequences from the immunoprecipitated complex, which was purified for downstream qPCR analysis.

### Statistical analysis

All statistical data were analyzed with SPSS 22.0 software. Data are shown as means ± SD or median and interquartile range. Statistical analysis was performed using t-tests or rank sum tests and all statistical tests were two-sided. *P* values ≤ 0.05 were considered significant.

## Results

### Differential expression of mRNAs in T (HbH-CS group) versus N (normal controls)

Group N included 3 male and 2 female cases, and group T had 2 male and 3 female cases. No statistically significant difference was observed in the gender between the two groups (*P* = 0.527). In addition, no statistically significant difference was observed in the age (years) between groups (32.00 ± 8.89 vs 28.20 ± 12.61, *P* = 0.597). S2a Fig in S1 File shows the volcano plot of m^6^A methylation-related enzymes. Heatmaps and scatter plots were used to visualise the differential expressions of mRNAs (S3 and S4a Figs in S1 File, respectively). METTL16 had the lowest P-value among all methylation-related enzymes. S3 Table in S1 File provides the top 25 differentially highly expressed mRNAs. We also observed that HBG 1 _ ENST00000330597 (fold change (FC) = 257.33, *P* = 0.0005), HBG 2 _ ENST00000336906 (FC = 5.35, *P* = 0.04) and HBG 2 _ ENST00000380252 (FC = 35.96, *P* = 0.006) showed significantly high expressions in patients with HbH-CS disease. The principal component analysis (PCA) diagram of mRNAs expression level, (S4c Fig in S1 File) revealed good differentiation between groups T and N. Gene Ontology (GO) analysis was performed statistically, and the results were plotted using the top GO package in R (S5 Fig in S1 File). Meanwhile, pathway analysis was performed using Fisher’s exact test. (S6 Fig in S1 File). S7 Fig in S1 File shows the protein–protein interaction (PPI) network of differentially expressed m^6^A-related enzymes. A coexpression relationship was observed between METTL16 and YTHDF3.

### Differential m^6^A-Methylated mRNAs, lncRNAs, and other small RNAs

Based on our m^6^A-mRNA and lncRNA epitranscriptomic microarray analysis, a total of 176 and 12,959 mRNAs were hypermethylated and hypomethylated in T versus N, respectively (S2b Fig in S1 File). Moreover, 51 and 3,186 lncRNAs were hypermethylated and hypomethylated in T versus N, respectively (S2c Fig in S1 File). Furthermore, 28 and 1,738 other small RNAs were hypermethylated and hypomethylated in T versus N, respectively (S2d Fig in S1 File). The default thresholds are FC≥ 1.5 or ≤ 1/1.5 and *p*-values ≤ 0.05. Herein, heatmaps were used to visualize deferentially methylated mRNAs, lncRNAs, and other small RNAs (S2e, S2f, S4b Figs in S1 File). The PCA diagram of mRNAs methylation level (S4d Fig in S1 File) revealed good differentiation between groups T and N. The results of the GSEA were shown in S7–S9 Files, the GOCC_ BLOOD_ MICROPARTICLE was significant *p* = 0.0001 (S2g Fig in S1 File) and its gene set contains haemoglobin globin related genes.

### Identification of hematological parameters related to mRNAs, lncRNAs, and other small RNAs in HbH-CS thalassemia patients

Pearson’s correlation analysis was used to determine the correlations between m^6^A-mRNA and lncRNA epitranscriptomic microarray expression level and methylation level and hematological parameters HbH, HbF, HbA2, HGB, MCV, MCH, MCHC, and SF in T. The genes whose absolute values of correlation coefficients with hemoglobin (HGB) and serum ferritin (SF) | Pearson R |>0.80 and *P* value < 0.05 were used for GO and KEGG analyses, and GO and KEGG analyses were performed on the metascape (S8a, S8b Fig in S1 File). The top 6 positive and negative HGB, SF-correlated mRNAs expression level, and methylation level are shown in the heatmap (S8c, S8d Fig in S1 File). Reader protein insulin like growth factor 2 mRNA binding protein 3 (IGF2BP3) showed a strong negative correlation with HGB in T (*P* < 0.001) and was for further verification. The positive and negative HbH, HbF, HbA2, HGB, MCV, MCH, MCHC, and SF-correlated mRNAs expression level, and methylation level, lncRNAs, miRNAs and other small RNAs methylation levels are shown in S2–S6 Files.

### Correlation analysis of m^6^A-related enzymes and various globins in microarray

The pearson correlation analysis of the expression levels of the major m^6^A-related enzymes is shown in the S9 Fig in S1 File, and the top two correlation coefficients of reading proteins with methylase METTL16_ENST00000263092 were IGF2BP3_ENST00000258729 R = 0.81 and *P* = 0.004 and YTHDF3_ENST00000623280 Pearson R = 0.74 and *P* = 0.01 (S9a Fig in S1 File). Pearson correlation analysis was carried out between the expression of various globins and the expression of m^6^A-related enzymes in T. The expression level of IGF2BP3_ENST00000258729 was positively correlated with that of HBG 2. The top 10 of the correlation coefficient of m^6^A-related enzymes are shown in the S9b Fig in S1 File. Pearson correlation analysis was carried out between the methylation level of various globins and the expression of m^6^A-related enzymes in T. The correlated m^6^A-related enzymes are shown in the S9c Fig in S1 File.

### Validate the microarray results in clinical samples

Clinical characteristics of participants. No significant differences were found in age (years) between the HbH group and the normal controls (21.97±12.10 vs 24.51±13.49, *P* = 0.350), and between the HbH-CS group and the normal controls (19.25±12.53 vs 24.51±13.49, *P* = 0.073). No significant differences were found in sex between the HbH group and the normal controls, *P* = 0.958, and between the HbH-CS group and the normal controls, *P* = 0.755. There were 14 males and 26 females in the HbH group, 20 males and 38 females in the normal control group, and 10 males and 22 females in the HbH-CS group.

**Fig 1:**
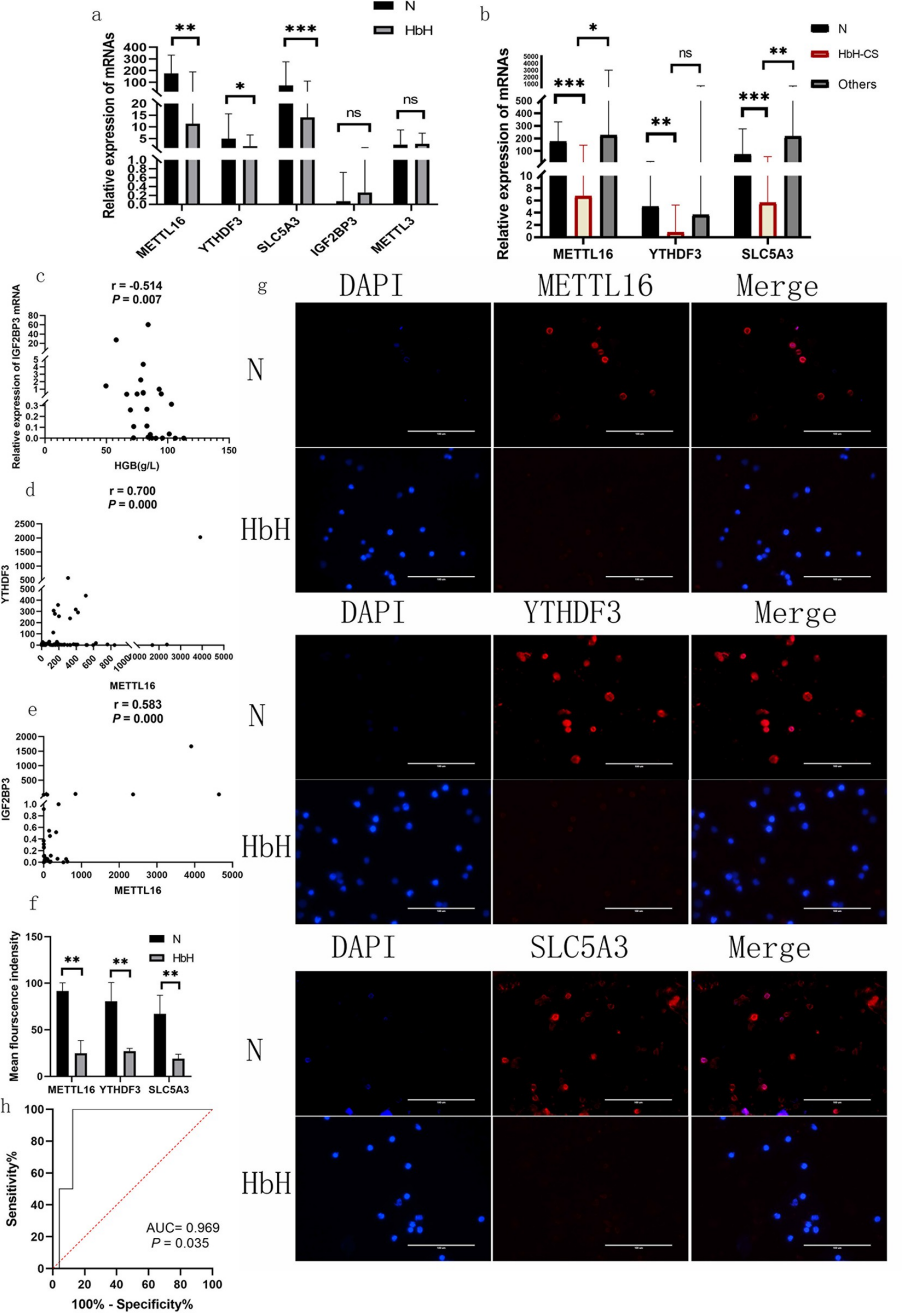
Validation and clinical significance of METTL16, YTHDF3 and IGF2BP3 in patients with HbH disease. METTL16, YTHDF3 and SLC5A3 mRNAs showed lower relative expressions in patients with HbH and HbH-CS diseases than those in normal controls without thalassemia (median with interquartile range) (a and b). Patients with HbH-CS disease presented lower relative expressions of METTL16 and SLC5A3 mRNAs than the patients with other types of HbH disease (—^SEA^/ α^WS^ α,—^SEA^/- α^3.7^ and—^SEA^/- α^4.2^) (median with interquartile range) (b). Spearman correlation analysis showed that the relative expression of IGF2BP3 mRNA was negatively correlated with HGB content in HbH-CS patients (c). METTL16 mRNA was positively correlated with YTHDF3 and IGF2BP3 mRNAs in all included subjects (d and e). Immunofluorescence showing that the protein expressions of METTL16, YTHDF3 and SLC5A3 were significantly downregulated in HbH patients compared with normal controls without thalassemia (magnification 400×) (mean with standard deviation (SD)) (f and g). The ROC curve demonstrates the potential diagnostic value of IGF2BP3 in the prediction of severe anaemia in HbH-CS disease (h). *P≤0.05, **P≤0.01 and *** *P≤*0.001.

Our research team found that METTL16 and YTH N6-methyladenosine RNA binding protein F3 (YTHDF3) were related to HbH-CS disease in previous studies [27]. In the transcriptome microarray data, we also found that the expression of m^6^A methyltransferase METTL16 (FC = 0.44, *P* < 0.001) was down-regulated in peripheral blood reticulocytes and nucleated erythrocytes of patients with HbH-CS disease. Hence, we continued to expand the samples and included 40 patients with HbH disease (32 patients—^ESA^/ α^CS^α patients, 2 cases—^SEA^/ α^WS^α, 5 cases—^SEA^/-α^3.7^, an example—^SEA^/-α^4.2^) and 58 normal controls without thalassemia. Then, we performed qRT-PCR and found that the relative expression of METTL16 and YTHDF3 mRNAs in patients with HbH and HbH-CS diseases was lower than that in normal controls without thalassemia (Fig 1a and 1b). The expression of METTL16 in patients with HbH-CS disease was lower than that in patients with other types of HbH disease (—^SEA^/ α^WS^ α,—^SEA^/- α^3.7^,—^SEA^/- α^4.2^) (Fig 1b). There was no significant difference in the expression level of IGF2BP3 mRNA between the HbH disease group and the normal control group (Fig 1a). In addition, the HGB (g/L) of patients with HbH-CS disease was lower than that of patients with other types of HbH disease (—SEA/ α^WS^ α,—SEA/- α^3.7^,—SEA/- α^4.2^) (80.82 ± 16.43 vs 109.17 ± 14.87, *P* = 0.000). The serum ferritin (ng/ml) of patients with HbH-CS disease was higher than that of patients with other types of HbH disease (—SEA/ α^WS^ α,—SEA/- α^3.7^,—SEA/- α^4.2^) (833.51 ± 1334.56 vs 497.40 ± 372.10, *P* = 0.552), but it was not statistically significant. Then, we detected IGF2BP3 mRNA using qRT-PCR in 26 HbH-CS patients. Correlation analysis showed that the expression of IGF2BP3 was negatively correlated with HGB content in HbH-CS patients (Fig 1c). Meanwhile, METTL16 mRNA was positively correlated with YTHDF3 and IGF2BP3 mRNAs (Fig 1d and 1e). We performed immunofluorescence on the peripheral blood CD71-positive cells of six patients with HbH disease and six normal controls without thalassemia. We found that the protein expression of METTL16 and YTHDF3 was significantly downregulated in HbH patients compared with normal controls without thalassemia. 4′,6-Diamidino-2-phenylindole (DAPI) staining showed that the majority of CD71-positive cells in the HbH group were not enucleated, and the majority of CD71-positive cells in the normal control group were enucleated (Figs 1f and 4g). We used the receiver operating characteristic (ROC) curve to measure the potential diagnostic value of insulin-like growth factor 2 mRNA-binding protein 3 (IGF2BP3) mRNA in the prediction of severe anaemia in HbH-CS disease. The area under the curve was 0.969 (95% confidence interval: 0.884–1.000, *P* = 0.035, Fig 1h).

### METTL16 protein expression decreased in K562 cells in a hemin environment

**Fig 2:**
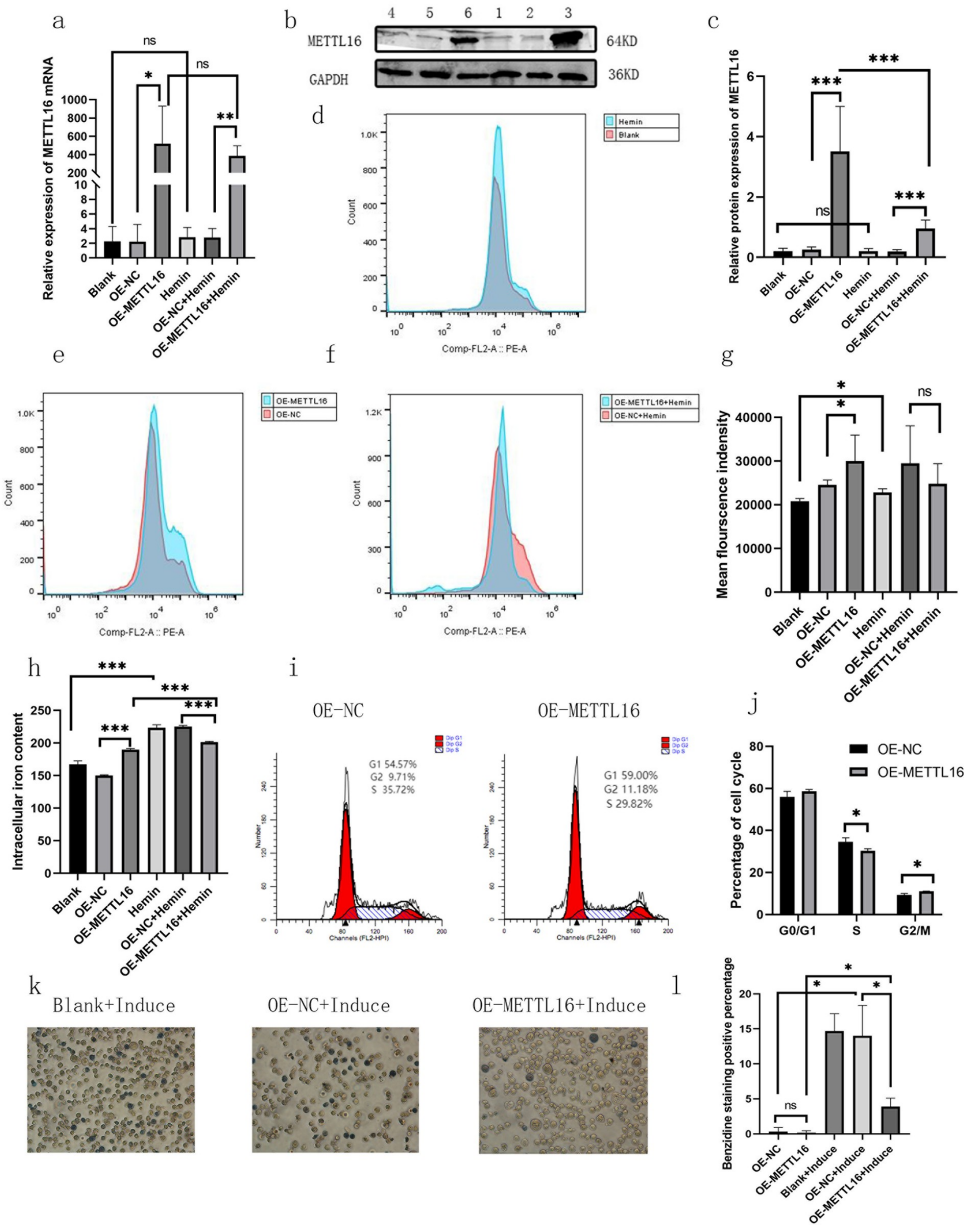
Biological function of METTL16. METTL16 mRNA (a) and protein (b and c) levels in K562 cells.1. Blank, 2. Overexpression (OE)—negative control (NC), 3. OE-METTL16, 4. hemin, 5. OE-NC+Hemin, and 6. OE-METTL16+Hemin. The ROS content of K562 cells overexpressing METTL16 was increased considerably. After 14 days of hemin treatment, the ROS content of K562 cells increased substantially, and no significant difference was observed in the ROS content between the empty-body and METTL16 overexpression groups (d–g). The iron content of K562 cells in different groups (h). K562 cells overexpressing METTL16 in the S phase decreased, the G2/M phase increased, and G0/G1 did not significantly change (I and j). After 5 days of induction with hemin, relatively few blue-stained cells overexpressed METTL16 (magnification 200×) (Fig 5k and 5l) (mean with SD). *P≤0.05, **P≤0.01 and *** *P≤*0.001.

Herein, lentivirus was used to stably overexpress METTL16. METTL16 mRNA and protein levels in K562 cells were considerably higher than those in empty vector control cells. After 14 days of treatment with iron-containing reagent hemin, the METTL16 mRNA level in K562 cells showed no notable difference compared with that before treatment. Hemin-treated K562 cells exhibited a slightly lower METTL16 protein expression level than those without treatment. The expression of METTL16 protein in cells overexpressing METTL16 with hemin treatment was notably lower than that in cells overexpressing METTL16 without hemin treatment (Fig 2a–2c).

### Effects of METTL16 overexpression on reactive oxygen species (ROS) and intracellular iron content in K562 cells

Flow cytometry was used to detect the ROS content of K562 cells. The ROS content of K562 cells overexpressing METTL16 was increased substantially. After 14 days of hemin treatment, the ROS content of K562 cells increased remarkably, and no notable difference was observed in the ROS content between the empty-body and METTL16-overexpression hemin treatment groups (Fig 2d–2g). The iron content of K562 cells was detected using an iron ion colorimetric assay kit. The iron content of K562 cells overexpressing METTL16 was increased considerably. In addition, after 14 days of hemin treatment, the iron content in K562 cells increased greatly, and the cellular iron content of the METTL16-overexpression hemin treatment group was reduced compared with the empty-vector hemin treatment group (Fig 2h).

### Overexpression of METTL16 altered the K562 cell cycle and prevented hemin induced hemoglobin production

Flow cytometry was used to detect the logarithmic growth phase K562 cell cycle. K562 cells overexpressing METTL16 in the S phase decreased, the G2/M phase increased, and G0/G1 did not significantly change (Fig 2i and 2j). After 14 days of hemin treatment, there was no significant difference in the cell cycle between K562 cells overexpressing METTL16 and the empty vector (S10 Fig in S1 File). The principle of benzidine staining is that the intracellular peroxidase can transfer the hydrogen atom of colorless 3,3-diaminobenzidine (DAB) to hydrogen peroxide, causing the former to catalyze the deposition of colored dyes at the site of the peroxidase in the cytoplasm. Hemoglobin or heme in red blood cells have peroxidase like activity, which can release new ecological oxygen from hydrogen peroxide and oxidize colorless benzidine to benzidine blue. We used hemin to treat the cells for 5 days, then stained them with benzidine. We found that after 5 days of induction with hemin, there were relatively few blue-stained cells with overexpression of METTL16 compared with the empty vector (Fig 2k and 2l).

### Search for possible reading proteins and target genes

**Fig 3:**
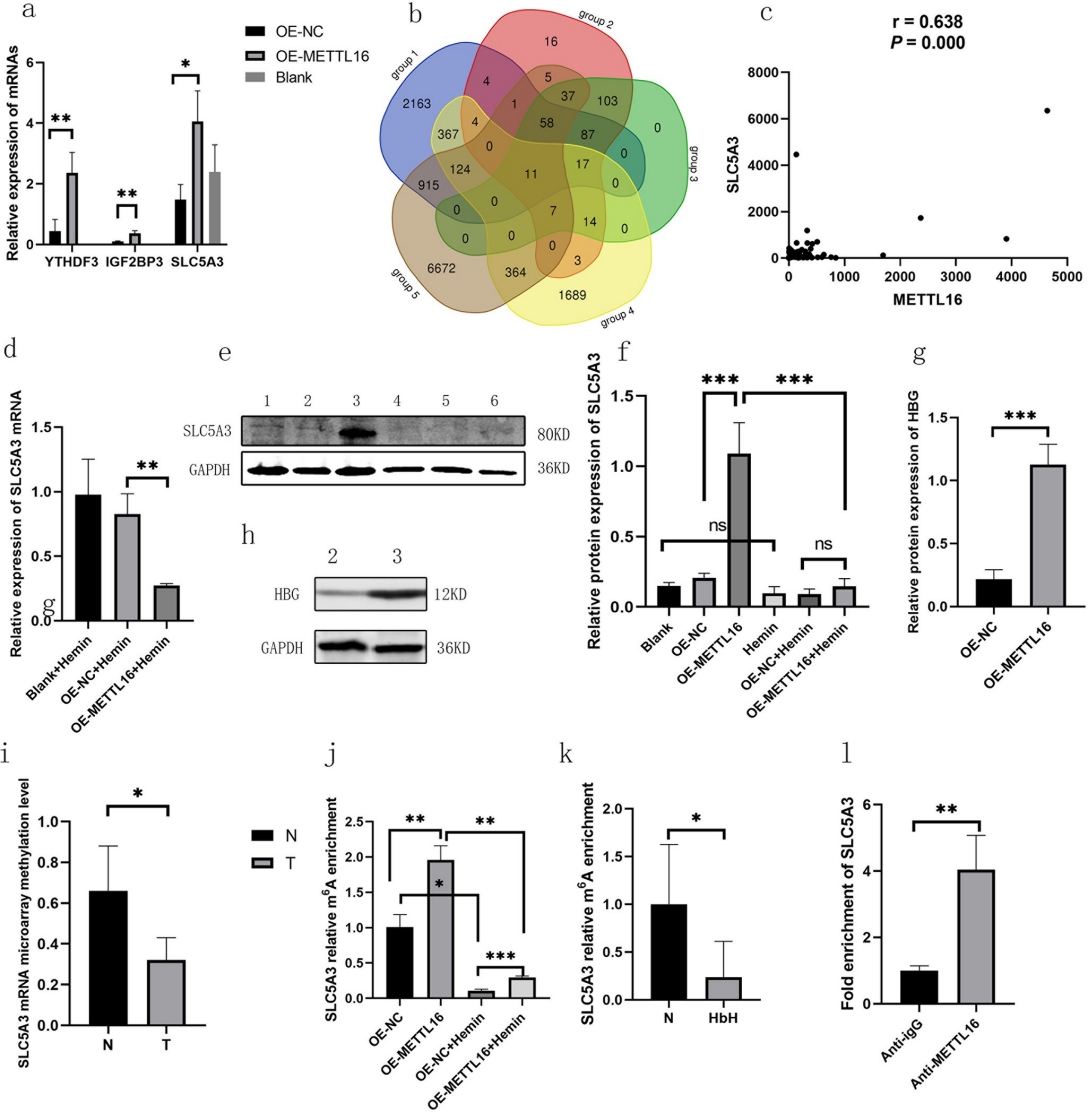
Search and validation of target genes. The expression levels of YTHDF3, IGF2BP3 and SLC5A3 mRNA in K562 cells overexpressing METTL16 were notably higher than those in the negative control (a). Venn diagram of METTL16 and YTHDF3 target genes (b). Pearson correlation analysis revealed the positive correlation of METTL16 with SLC5A3 in all the subjects involved (c). After the treatment of K562 cells with iron-containing reagent hemin for 14 days, the level of SLC5A3 mRNA in K562 cells overexpressing METTL16 was lower than that in the empty-vector control (d). The expression of SLC5A3 protein of K562 cells in different groups. 1. Blank, 2. OE-NC, 3. OE-METTL16, 4. Hemin, 5. OE-NC+Hemin, and 6. OE-METTL16+Hemin (e and f). Expression of HBG protein of K562 cells in different groups. 2. OE-NC. 3. OE-METTL16 (g and h). In the chip data, SLC5A3 showed hypomethylation in the HbH-CS group (i). The methylation level of the SLC5A3 target site in K562 cells overexpressing METTL16 was remarkably higher than that in the empty vector control. After 14 days of hemin treatment, the methylation level of SLC5A3 target site was reduced considerably compared with that before treatment (j). The methylation level of SLC5A3 in HbH patients was notably lower than that in normal controls (k). In the RIP experiment on K562 cells overexpressing METTL16, the RNA enriched by the METTL16 antibody was four times that of IgG (l). (mean with SD). *P≤0.05, **P≤0.01 and *** *P≤*0.001.

The mRNA expression of YTHDF3 and IGF2BP3 was upregulated by the overexpression of METTL16, as depicted in Fig 3a. Therefore, YTHDF3 and IGF2BP3 may serve as the RNA-binding proteins responsible for recognizing and interacting with METTL16. In this study, to look for the target genes of METTL16 and YTHDF3, we determined the correlation between METTL16 expression and the methylation level of all mRNAs in the chip. We categorized mRNAs with r > 0.8 and *P* < 0.05 as group 1; and correlation analysis was conducted between METTL16 expression and mRNA with differential expression in the microarray. Meanwhile, mRNA with r > 0.8 and *P* < 0.05 were taken as group 2; the expression level of YTHDF3_ENST00000623280 was associated with the mRNA with differentially expression in the chip, and the mRNA with r> 0.8 and *P* < 0.05 was used as group 3; the reactive oxygen species biosynthetic process related genes were downloaded from GeneCards as group 4; the potential targets obtained by YTHDF3 was found through RIP seq on the M6A2Target website as group 5 and intersect the above five groups. Finally, 11 genes including MBP, LYN, SLC5A3, NFKB1, IGF2R, PFKFB3, ANKFY1, IL6R, VEGFA, MYC, and RIPK1 were obtained (Fig 3b). To find the specific genes regulated by METTL16 and IGF2BP3, through bioinformatics analysis, we observed that haemoglobin subunit gamma-1 (HBG1) was significantly overexpressed in the HbH-CS group, with the largest differential multiple among all differentially expressed globin peptide chain genes. Correlation analysis revealed a positive correlation between the expressions of IGF2BP3_ENST00000258729 and ENST00000336906 _ HBG 2. We searched for HBG1 mRNA and HBG2 mRNA-predicted RNA-binding proteins in the POSTAR3 database and discovered that HBG1 mRNA- and HBG2 mRNA-predicted RNA binding proteins were IGF2BP3.

### Verify target gene and reading protein

We performed qRT-RCR and found that the expression level of SLC5A3 mRNA in K562 cells overexpressing METTL16 was significantly higher than that in the negative control (Fig 3a). In the chip data, SLC5A3 (FC = 0.04, *P* < 0.001) and METTL16 were down-regulated in the peripheral blood nucleated erythrocytes and reticulocytes of HbH-CS patients. A qRT-PCR was performed in 40 patients with HbH disease (32 patients—^ESA^/ α^CS^α patients, 2 cases—^SEA^/ α^WS^α, 5 cases—^SEA^/-α^3.7^, an example—^SEA^/-α^4.2^) and 58 normal controls without thalassemia. We found that the relative expression of SLC5A3 mRNA in patients with HbH disease and HbH-CS disease was lower than that in normal controls without thalassemia, and the relative expression of SLC5A3 mRNA in patients with HbH-CS disease was lower than that in patients with other types of HbH disease (—^SEA^/ α^WS^ α,—^SEA^/- α^3.7^,—^SEA^/- α^4.2^) (Fig 1a and 1b). Pearson correlation analysis found that METTL16 mRNA was positively correlated with SLC5A3 mRNA in all the subjects involved (Fig 3c). We also found that YTHDF3 mRNA was positively correlated with SLC5A3 mRNA in all the subjects involved (r = 0.537, *P* = 0.000). We performed immunofluorescence on the peripheral blood CD71-positive cells of six patients with HbH disease and six normal controls without thalassemia. We found that the protein expression of SLC5A3 was significantly downregulated in HbH patients compared with normal controls without thalassemia (Fig 1f and 1g). After treatment of K562 cells with iron-containing reagent hemin for 14 days, the level of SLC5A3 mRNA in K562 cells overexpressing METTL16 was lower than that in the empty vector control (Fig 3d). Meanwhile, the expression of solute carrier family 5 member 3 (SLC5A3) protein in K562 cells overexpressing METTL16 without hemin treatment was notably higher than that in the empty vector control. After 14 days of hemin treatment, the expression of SLC5A3 protein in K562 cells slightly decreased compared with that before hemin treatment. The cells overexpressing METTL16 with hemin treatment exhibited substantially lower expression.

SLC5A3 protein in compared with that in cells overexpressing METTL16 without hemin treatment. After 14 days of hemin treatment, no substantial difference was found in the expression of SLC5A3 protein in K562 cells overexpressing METTL16 with hemin treatment compared with the empty vector control with hemin treatment (Fig 3e and 3f). The expression level of HBG protein in K562 cells overexpressing METTL16 without hemin treatment increased remarkably compared with that of the empty vector control without hemin treatment (Fig 3g and 3h).

**Fig 4:**
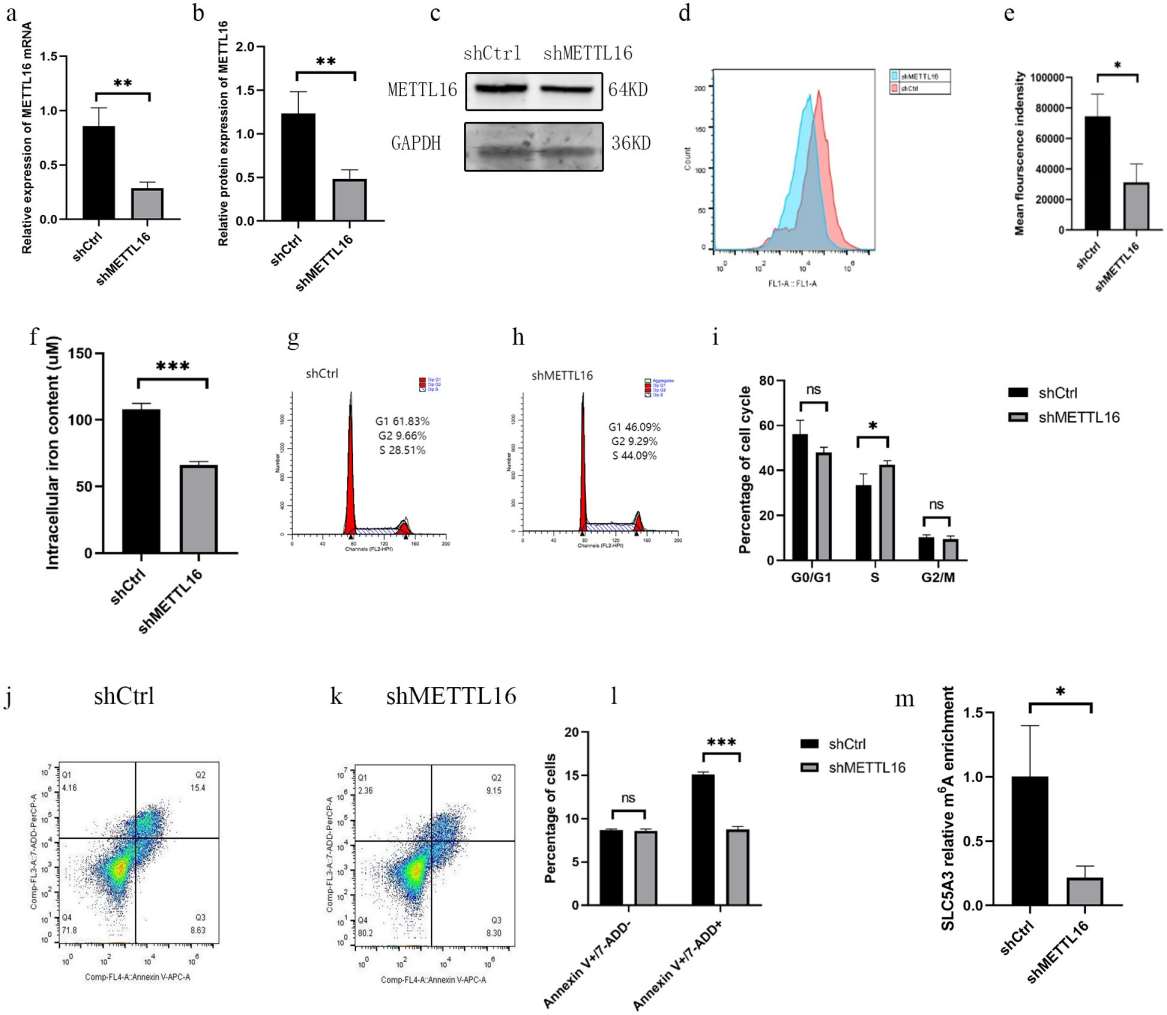
Knocking down METTL16 on K562 cells. Expression of METTL16 mRNA (a) and protein (b, c) levels after METTL16 knockdown on K562 cells. Changes in ROS levels after knockdown of METTL16 in K562 cells. (d, e). Iron content of K562 cells knocked down by METTL16 was significantly lower than that in the control group (f). Knock down of METTL16 altered the K562 cell cycle (g-i). Effects of knockdown of METTL16 on apoptosis and cell rupture in K562 cells (j-l). Methylation level of SLC5A3 target site in METTL16 knockdown K562 cells was significantly lower than that in the control group (m). *P≤0.05, **P≤0.01 and *** *P≤*0.001.

We used lentivirus to stably knockdown METTL16 in K562 cells. METTL16 mRNA and protein levels decreased compared with those in the control cells (Fig 4a–4c). The ROS (Fig 4d and 4e) and intracellular iron contents (Fig 4f) of METTL16 knockdown K562 cells were reduced considerably compared with those of the control group. K562 cells with knockdown of METTL16 increased the number of S-phase cells compared with the controls (Fig 4j–4i). Knockdown of METTL16 in K562 cells resulted in a considerable decrease in AnnexinV-APC- and 7-AAD-positive cells compared with the control group (Fig 4j–4l).

**Fig 5:**
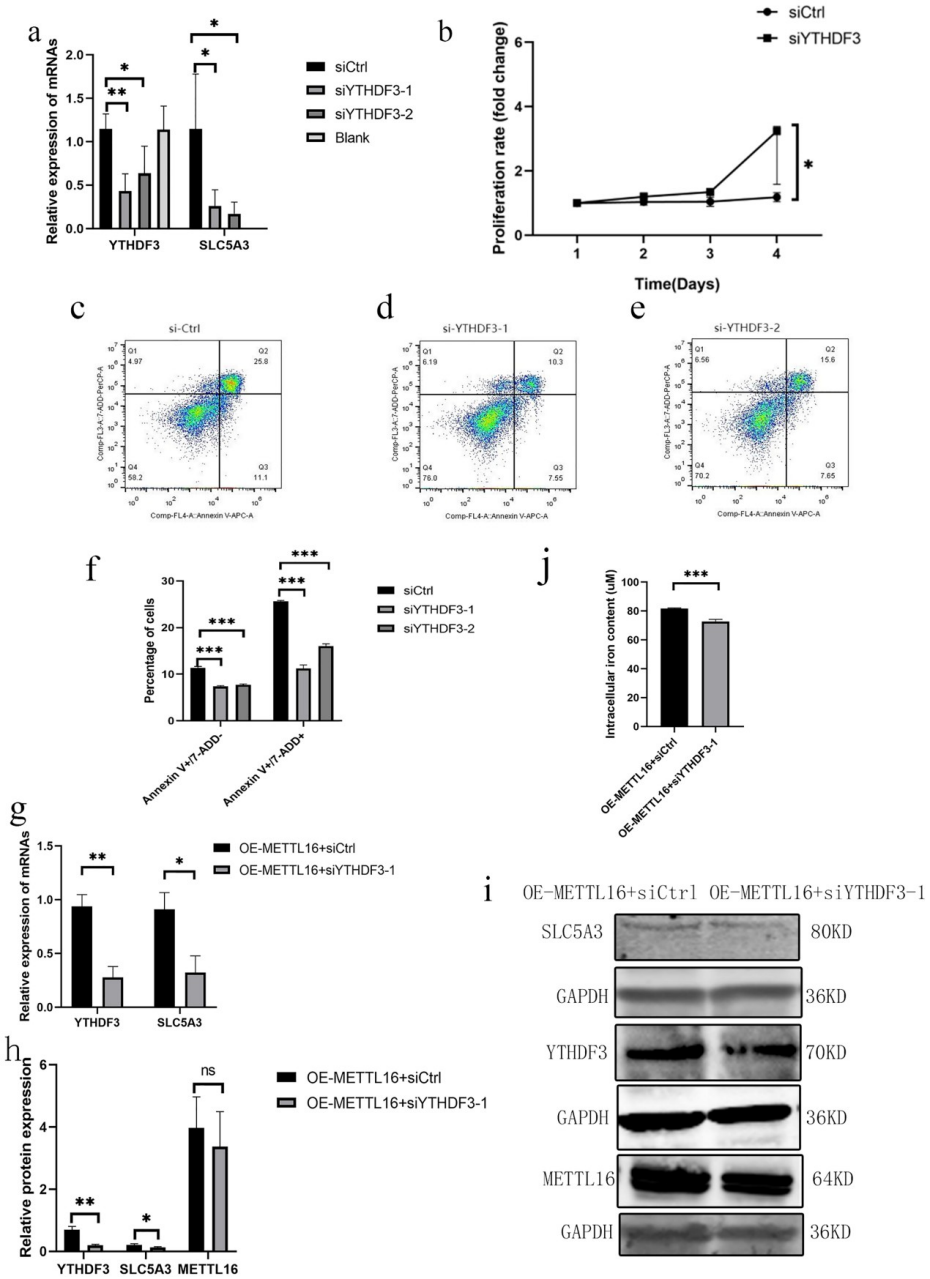
Knocking down YTHDF3 in K562 cells and K562 cells overexpressing METTL16. Expression of YTHDF3 and SLC5A3 mRNAs after knockdown of YTHDF3 in K562 cells (mean with SD) (a). K562 cells with YTHDF3 knockdown showed increased proliferative capacity (median with interquartile range) (b). Effects of knockdown of YTHDF3 on apoptosis of K562 cells (mean with SD) (c-f). Knockdown of YTHDF3 rescued the increase in SLC5A3 mRNA and protein levels caused by the overexpression of METTL16. In K562 cells overexpressing METTL16, YTHDF3 and SLC5A3 mRNA (g) and protein levels (h and i) were significantly reduced compared with the control group (mean with SD). Effect of simultaneous knockdown of YTHDF3 in K562 cells overexpressing METTL16 on their intracellular iron content (mean with SD) (j). *P≤0.05, **P≤0.01 and *** *P≤*0.001.

We used two siRNA and siCtrl to transfect K562 cells. Verification by quantitative reverse-transcription–polymerase chain reaction (qRT-PCR) at 72 h posttransfection revealed that siYTHDF3-1 and siYTHDF3-2 decreased YTHDF3 and SLC5A3 mRNA expressions (Fig 5a). Knockdown of YTHDF3 in K562 cells increased their proliferation ability (Fig 5b) and reduced cell apoptosis (Fig 5c–5f).

In this study, we conducted the simultaneous knockdown of YTHDF3 in K562 cells overexpressing METTL16. Our results showed a considerable reduction in the mRNA and protein levels of YTHDF3 and SLC5A3 compared with the knockdown control group of K562 cells overexpressing METTL16 (Fig 5g–5i). Furthermore, we observed a decrease in ROS content upon simultaneous knockdown of YTHDF3 in K562 cells overexpressing METTL16, although the difference was not statistically significant (S11 Fig in S1 File). In addition, we observed that knockdown of YTHDF3 simultaneously in K562 cells overexpressing METTL16 resulted in a decrease in intracellular iron content compared with the knockdown of the control group in K562 cells overexpressing METTL16 (Fig 5j).

In the chip data, SLC5A3 showed hypomethylation in the HbH-CS group (FC = 0.47, *P* = 0.013) (Fig 3i). Expression of METTL16 was positively correlated with SLC5A3 methylation level (r = 0.8, *P* = 0.005) and expression level (r = 0.87, *P* = 0.001). The methylation level of the SLC5A3 target site in K562 cells overexpressing METTL16 was significantly higher than that in the empty vector control. After 14 days of hemin treatment, the methylation level of the SLC5A3 target site was significantly reduced compared to before treatment (Fig 3j). Knocking down METTL16 significantly reduces the methylation level of the SLC5A3 target site in K562 cells compared to the control (Fig 4m). We verified the methylation level of the SLC5A3 target site in eight patients with HbH disease and four normal controls. The methylation level of SLC5A3 in HbH patients was significantly lower than that in normal controls (Fig 3k). Meanwhile, in the RIP experiment on K562 cells overexpressing METTL16, the RNA enriched by the METTL16 antibody was four times that of the IgG (Fig 3l). The METTL16 antibody enrichment group can be significantly enriched in the METTL16 protein bands. However, no METTL16 protein bands were seen in the IgG negative control group (S12 Fig in S1 File).

## Discussion

This study found that METTL16, YTHDF3, and SLC5A3 mRNAs were downregulated in HbH patients. Solid carrier family 5 member 3 (SLC5A3) is one of the three known inositol transporters, it belongs to the solute carrier family 5 and is also known as sodium-coupled Myo inositol transporter, SMIT and it is widely expressed in various tissues [30]. SLC5A3 predicted to act upstream of or within several processes including positive regulation of reactive oxygen species biosynthetic process and regulation of respiratory gaseous exchange. [It was provided by Alliance of Genome Resources, Apr 2022.] In vivo, it participates in oxidative stress and positive regulation of reactive oxygen species (ROS) biosynthetic process, inflammation, inositol transport, regulation of osmotic pressure, ion channels, cell proliferation and apoptosis, vasoconstriction, and so on. Diseases involved in the study include neuropsychiatric diseases (schizophrenia and Parkinson’s disease) [31, 32], diseases of the musculoskeletal system (osteoarthritis, myositis, and lumbar disc herniation) [33–35], endocrine diseases (polycystic ovary syndrome, diabetes, and obesity) [36, 37], acute renal injury [38], non-small cell lung cancer [39], and so on. In the field of blood system diseases, there are studies primarily on acute myeloid leukemia. SLC5A3 protein is crucial for leukemia cells with high nutritional requirements and a relative lack of inositol [30]. A study involving the genotype and clinical test characteristics of 595 children with HbH disease found that the degree of anemia in children with HbH-CS was more severe than other types of HbH [40]. We also found that the degree of anemia in patients with HbH-CS disease was more serious than that in patients with other types of HbH disease (—^SEA^/ α^WS^ α,—^SEA^/- α^3.7^,—^SEA^/- α^4.2)^, which was consistent with previous studies [40, 41]. We found that the serum ferritin of patients with HbH-CS disease was higher than that of patients with other types of HbH disease (—^SEA^/ α^WS^ α,—^SEA^/- α^3.7^,—^SEA^/- α^4.2^), but it was not statistically significant, probably because of the small sample size. This study also found that METTL16 and SLC5A3 mRNA were down-regulated in HbH-CS disease patients compared with other types of HbH disease patients (—^SEA^/ α^WS^ α,—^SEA^/- α^3.7^,—^SEA^/- α^4.2^). It may be because due to genotype differences, patients with HbH-CS disease are more anaemic, and ineffective hematopoiesis and iron overload are more serious, resulting in compensatory METTL16 and SLC5A3 decline reducing excessive iron entry into cells and reducing the increase of ROS caused by iron overload. Thereby suggesting that m^6^A methylation modification may be related to the severity of the disease in patients with HbH disease.

In this study, the expression of SLC5A3 and the content of ROS in K562 cells overexpressing METTL16 significantly increased. The ROS content of METTL16 knockdown K562 cells was significantly reduced. The methylation level of the SLC5A3 target site in K562 cells overexpressing METTL16 was significantly higher than that in the empty vector control, knocking down METTL16 significantly reduces the methylation level of the SLC5A3 target site in K562 cells compared to the control, thereby suggesting that SLC5A3 may be the target gene of METTL16. Moreover, METTL16 can play a role by regulating the m^6^A modification level of SLC5A3 mRNA. We speculate that METTL16 can affect the protein expression of SLC5A3 mRNA by changing its m^6^A modification level, and regulating the biological processes such as oxidative stress and ROS synthesis of K562 cells. After 14 days of hemin treatment, the expression of METTL16 and SLC5A3 proteins in K562 cells slightly decreased. At the same time, K562 cells overexpressing METTL16 were treated with hemin, and the results showed that after 14 days of hemin treatment, the expressions of METTL16 and SLC5A3 proteins were significantly lower than that before hemin treatment. In addition, the methylation level of the SLC5A3 target site significantly decreased. After 14 days of hemin treatment, no significant difference was found in ROS content between the empty body and the METTL16 overexpression group. The expression of SLC5A3 protein in K562 cells overexpressing METTL16 with hemin treatment had no significant difference compared with the empty vector control with hemin treatment. SLC5A3 expression is regulated by the content of iron in cells. The expression of SLC5A3 in iron-deposited cells is down-regulated, which reduces the inositol entry into cells, and plays a role in stabilizing the osmotic pressure in cells [42]. Hence, we believe that high iron can lead to the down-regulation of the METTL16 protein level, thus leading to the down-regulation of the SLC5A3 mRNA m^6^A methylation level. It also leads to the decrease of SLC5A3 protein level, which may be a compensatory reaction of cells to reduce the damage caused by oxidative stress caused by high iron. Oxidative damage by reactive oxygen species is one of the main contributors to cell injury and tissue damage in thalassemia patients [43]. In the pathogenesis of HbH disease, iron deposition in red blood cells leads to the increase of ROS, and oxidative stress leads to cell damage, which is an important link leading to and aggravating the destruction of red blood cells, hemolysis, and anemia [44]. Moreover, SLC5A3 can regulate ROS synthesis and oxidative stress, thus, we speculate that SLC5A3 has a potential regulatory effect on the pathophysiological process and clinical manifestations of HbH. The downregulation of METTL16 and SLC5A3 mRNA in HbH disease patients may be a compensatory response, which can reduce the elevated ROS caused by iron deposition and oxidative stress. This study also found that the iron content in K562 cells overexpressing METTL16 was significantly increased. After 14 days of hemin treatment, the iron content in K562 cells significantly increased. Furthermore, the intracellular iron content of the METTL16 overexpression hemin treatment group was lower than that of the empty vector hemin treatment group. Thus, we speculated that SLC5A3 might also transport iron in the process of inositol transport. After METTL16 overexpression, the expression of SLC5A3 also increased, thereby leading to a significant increase in intracellular iron content. After 14 days of hemin treatment, the level of SLC5A3 mRNA in K562 cells overexpressing METTL16 decreased compared with the empty vector control, and no significant difference was found in the expression of SLC5A3 protein in K562 cells overexpressing METTL16 with hemin treatment compared with the empty vector control with hemin treatment, which may lead to the reduction of iron content in cells of METTL16 overexpressing group with hemin treatment compared with the empty vector group with hemin treatment.

It has been reported that YTH N6-methyladenosine RNA binding protein F3 (YTHDF3)-mediated PRDX3 translation can regulate mitochondrial ROS level [45]. We found that overexpression of METTL16 increases the mRNA expression of the reading protein YTHDF3 and ROS level. METTL16 was positively correlated with YTHDF3 in all subjects included. Thus, it suggests that YTHDF3 may be the reading protein of METTL16. The present study demonstrated that the knockdown of YTHDF3 facilitated the proliferation of K562 cells, and its low expression occurred in patients with HbH disease, which is consistent with the compensatory hematopoietic performance of such patients. Knockdown of YTHDF3 or METTL16 in K562 cells reduced cell apoptosis or rupture. This study revealed the downregulation of METTL16 and YTHDF3 in patients with HbH disease, possibly due to the compensatory response of the body to reduce the aggregation of HbH inclusion bodies on the red blood cell membrane, which leads to a decrease in the plasticity of red blood cells. Furthermore, our investigation revealed that the simultaneous knockdown of YTHDF3 in K562 cells overexpressing METTL16 resulted in a nonsignificant reduction in ROS content compared with the knockdown control group. The possible reason is that the overexpression of METTL16 led to an increased ROS content in K562 cells, which is not only dependent on the reading protein YTHDF3 but may also be caused by other mechanisms. Moreover, after 14 days of treatment with iron-containing reagent hemin, the level of SLC5A3 mRNA in K562 cells overexpressing METTL16 was lower than that in the empty vector control. The possible reason is that reading protein YTHDF3 not only regulates RNA stability [46, 47] and mRNA translation efficiency, but also mediates mRNA degradation through direct interaction with YTHDF2 [48, 49].

DNA synthesis occurs during the S phase of the cell cycle, and the G2/M checkpoint is one of the key checkpoints, which determines whether or not a cell can enter mitosis. The cell cycle stops when DNA damage occurs in this phase, thereby resulting in G2/M phase arrest. If the damaged cells fail to repair the damaged DNA, they will enter apoptosis [50]. The S phase cells of K562 cells overexpressing METTL16 decreased, and the G2/M phase cells increased. K562 cells with knockdown METTL16 increased S phase cells compared to control. Hence, it is speculated that overexpression of METTL16 may lead to an increase in ROS content in cells, thus leading to cell cycle arrest [51].

This study found that the expression of IGF2BP3 was negatively correlated with HGB content in HbH-CS patients, thereby suggesting that the expression levels of the m^6^A-related enzymes may be related to the severity of HbH-CS disease. We found that IGF2BP3 has a good predictive value for severe anemia in HbH-CS disease by plotting ROC curves, suggesting that IGF2BP3 may be a biomarker for predicting the severity of HbH-CS disease. This study also found that overexpression of METTL16 can reduce hemin-induced hemoglobin production. Overexpression of METTL16 increased the mRNA expression of the reading protein IGF2BP3. METTL16 was positively correlated with IGF2BP3 in all subjects included, thereby suggesting that IGF2BP3 may be the reading protein of METTL16. This study also found that the expression of IGF2BP3_ENST00000258729 was positively correlated with the expression of HBG2 in patients with HbH-CS disease through the analysis of bioinformatics. HBG were highly expressed in HbH-CS patients. The expression level of HBG protein in K562 cells overexpressing METTL16 without hemin treatment was significantly increased compared to the empty body control without hemin treatment. The IGF2BP (insulin-like growth factor 2 mRNA-binding proteins) family of proteins can maintain their stability and induce translation by specifically binding to the m^6^A to modify the mRNA [52]. Elagib et al. [53] found that IGF2BP3 neonatal expression regulates the human fetal-adult megakaryocyte transition. Meanwhile, Tangprasittipap et al. [54] found that the expression of IGF2BP3 in fetal liver tissue was up-regulated compared with adult peripheral red blood cells, and it could be identified as a new regulator of the transition from fetal to adult erythroid state. De Vasconcellos et al. found that among cultured primary erythroblasts, both IGF2BP1 and IGF2BP3 are expressed in cord blood erythroblasts but at background levels in adult blood erythroblasts [55]. HBG is normally expressed mainly during fetal life. A switch from γ-globin (HBG) to β-globin expression begins before birth and completes by the time the baby reaches 6 months of age [2]. Therefore, it can be inferred that IGF2BP3 may participate in hemoglobin synthesis. The production of tetramers of γ-chains (γ4) was known as haemoglobin Bart’s. Hb Bart’s poor ability to transport oxygen leads to hypoxia in tissues and organs of the whole body. This results in a progressive severe anemia [56]. We speculated that METTL16 may alter the expression level of HBG by reading the protein IGF2BP3, which may improve the symptoms of HbH-CS patients.

## Conclusion

**Fig 6:**
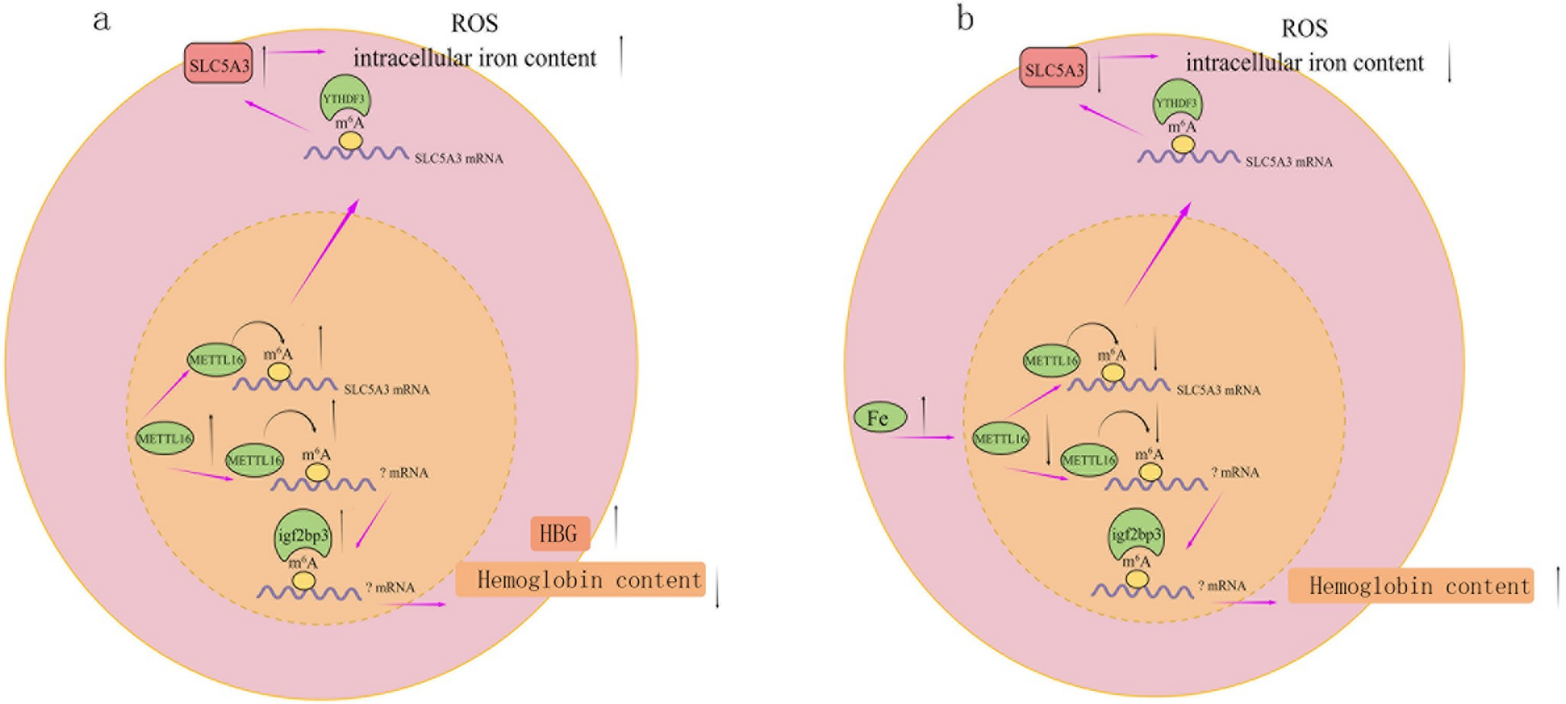
Schematic summarising our findings. (a) Overexpression of METTL16. (b) Cells overexpressing METTL16 were treated with hemin for 14 days (this figure was drawn by Figdraw).

In conclusion, this study revealed that METTL16 can alter the m^6^A modification level of SLC5A3 mRNA in high-iron or non-high iron environment. YTHDF3 may read the m^6^A modification information of SLC5A3 mRNA, affect its protein expression regulate ROS synthesis and intracellular iron content in red blood cells and thus participate in the pathological and physiological processes of HbH disease. Moreover, METTL16 possibly affects the expression of haemoglobin through IGF2BP3, which results in regulated clinical phenotype of HbH-CS disease. Fig 6 depicts the full-text summary mechanism diagrams. However, these findings need to be verified by additional mechanism research and animal experiments. Given the highly heterogeneous clinical phenotype of HbH-CS disease, prenatal diagnosis and genetic counselling may cause confusion. IGF2BP3 expression can be used as indicators to predict the clinical phenotype of HbH-CS disease and evaluate its severity. This has a good guiding significance for genetic counselling. Furthermore, monitoring and management should be strengthened for HbH-CS patients with elevated IGF2BP3. We can also develop drugs for the METTL16_ YTHDF3_ SLC5A3 pathway, such as agents designed to regulate METTL16 to reduce ROS and change the expression of IGF2BP3 to improve haemoglobin, which will provide a new direction for the treatment of HbH disease.

## Supporting information

S1 File Supplementary information. (DOCX)

S2 File mRNAs expression level. (XLSX)

S3 File mRNAs methylation level. (XLSX)

S4 File lncRNAs methylation level. (XLSX)

S5 File miRNAs methylation level. (XLSX)

S6 File Other small RNAs methylation level. (XLSX)

S7 File mRNA methylation level GSEA-GOBP. (XLSX)

S8 File mRNA methylation level GSEA-GOCC. (XLSX)

S9 File mRNA methylation level GSEA-GOMF. (XLSX)

S1 Raw images (PDF)

## Abbreviations

FC: Fold change
GO: Gene Ontology
GSEA: gene set enrichment analysis
HBG: γ-globin gene
HbH: hemoglobin H
HbH-CS: hemoglobin H-Constant Spring
HGB: hemoglobin
IGF2BP3: insulin like growth factor 2 mRNA binding protein 3
K562 cells: Human chronic myeloid leukemia cells
KEGG: Kyoto Encyclopedia of Genes and Genomes
lnc RNA: Long non-coding RNA
m^6^A: N6-methyladenosine
m^6^A: N6-methyladenosine
METTL16: methyltransferase 16
mRNA: messenger RNA
PCA: Principal Component Analysis
qRT-PCR: Real-time quantitative PCR
ROS: Reactive oxygen species
SF: serum ferritin
SLC5A3: solute carrier family 5 member 3
YTHDF3: YTH N6-methyladenosine RNA binding protein F3

## References

[1] Piel FB, Weatherall DJ. The α-thalassemias. N Engl J Med. 2014;371(20):1908–16. Epub 2014/11/13. doi: 10.1056/NEJMra1404415 . 25390741

[2] Taher AT, Weatherall DJ, Cappellini MD. Thalassaemia. Lancet. 2018;391(10116):155–67. Epub 2017/08/05. doi: 10.1016/S0140-6736(17)31822-6 . 28774421

[3] Harteveld CL, Higgs DR. Alpha-thalassaemia. Orphanet J Rare Dis. 2010;5:13. Epub 2010/05/29. doi: 10.1186/1750-1172-5-13 . 20507641 PMC2887799

[4] Tamary H, Dgany O. Alpha-Thalassemia. In: Adam MP, Everman DB, Mirzaa GM, Pagon RA, Wallace SE, Bean LJH, et al., editors. GeneReviews(^®^). Seattle (WA): University of Washington, Seattle Copyright © 1993–2022, University of Washington, Seattle. GeneReviews is a registered trademark of the University of Washington, Seattle. All rights reserved.; 1993.

[5] Ekwattanakit S, Siritanaratkul N, Viprakasit V. A prospective analysis for prevalence of complications in Thai nontransfusion-dependent Hb E/β-thalassemia and α-thalassemia (Hb H disease). Am J Hematol. 2018;93(5):623–9. Epub 2018/01/24. doi: 10.1002/ajh.25046 . 29359464

[6] Rivella S. Ineffective erythropoiesis and thalassemias. Curr Opin Hematol. 2009;16(3):187–94. Epub 2009/03/26. doi: 10.1097/MOH.0b013e32832990a4 . 19318943 PMC3703923

[7] Todkari IA, Chandrasekaran AR, Punnoose JA, Mao S, Haruehanroengra P, Beckles C, et al. Resolving altered base-pairing of RNA modifications with DNA nanoswitches. Nucleic Acids Res. 2023;51(20):11291–7. Epub 2023/10/09. doi: 10.1093/nar/gkad802 . 37811879 PMC10639047

[8] Tang C, Klukovich R, Peng H, Wang Z, Yu T, Zhang Y, et al. ALKBH5-dependent m6A demethylation controls splicing and stability of long 3’-UTR mRNAs in male germ cells. Proc Natl Acad Sci U S A. 2018;115(2):E325–e33. Epub 2017/12/28. doi: 10.1073/pnas.1717794115 . 29279410 PMC5777073

[9] Bartosovic M, Molares HC, Gregorova P, Hrossova D, Kudla G, Vanacova S. N6-methyladenosine demethylase FTO targets pre-mRNAs and regulates alternative splicing and 3’-end processing. Nucleic Acids Res. 2017;45(19):11356–70. Epub 2017/10/05. doi: 10.1093/nar/gkx778 . 28977517 PMC5737695

[10] Zhou J, Wan J, Shu XE, Mao Y, Liu XM, Yuan X, et al. N(6)-Methyladenosine Guides mRNA Alternative Translation during Integrated Stress Response. Mol Cell. 2018;69(4):636–47.e7. Epub 2018/02/13. doi: 10.1016/j.molcel.2018.01.019 . 29429926 PMC5816726

[11] Vu LP, Pickering BF, Cheng Y, Zaccara S, Nguyen D, Minuesa G, et al. The N(6)-methyladenosine (m(6)A)-forming enzyme METTL3 controls myeloid differentiation of normal hematopoietic and leukemia cells. Nat Med. 2017;23(11):1369–76. Epub 2017/09/19. doi: 10.1038/nm.4416 . 28920958 PMC5677536

[12] Barbieri I, Tzelepis K, Pandolfini L, Shi J, Millán-Zambrano G, Robson SC, et al. Promoter-bound METTL3 maintains myeloid leukaemia by m(6)A-dependent translation control. Nature. 2017;552(7683):126–31. Epub 2017/12/01. doi: 10.1038/nature24678 . 29186125 PMC6217924

[13] Zhao Y, Shi Y, Shen H, Xie W. m(6)A-binding proteins: the emerging crucial performers in epigenetics. J Hematol Oncol. 2020;13(1):35. Epub 2020/04/12. doi: 10.1186/s13045-020-00872-8 . 32276589 PMC7146974

[14] Yu R, Li Q, Feng Z, Cai L, Xu Q. m6A Reader YTHDF2 Regulates LPS-Induced Inflammatory Response. Int J Mol Sci. 2019;20(6). Epub 2019/03/17. doi: 10.3390/ijms20061323 . 30875984 PMC6470741

[15] Mendel M, Chen KM, Homolka D, Gos P, Pandey RR, McCarthy AA, et al. Methylation of Structured RNA by the m(6)A Writer METTL16 Is Essential for Mouse Embryonic Development. Mol Cell. 2018;71(6):986–1000.e11. Epub 2018/09/11. doi: 10.1016/j.molcel.2018.08.004 . 30197299 PMC6162343

[16] Wang HF, Kuang MJ, Han SJ, Wang AB, Qiu J, Wang F, et al. BMP2 Modified by the m(6)A Demethylation Enzyme ALKBH5 in the Ossification of the Ligamentum Flavum Through the AKT Signaling Pathway. Calcif Tissue Int. 2020;106(5):486–93. Epub 2020/01/04. doi: 10.1007/s00223-019-00654-6 . 31897529

[17] Pendleton KE, Chen B, Liu K, Hunter OV, Xie Y, Tu BP, et al. The U6 snRNA m(6)A Methyltransferase METTL16 Regulates SAM Synthetase Intron Retention. Cell. 2017;169(5):824–35.e14. Epub 2017/05/20. doi: 10.1016/j.cell.2017.05.003 . 28525753 PMC5502809

[18] Ruszkowska A, Ruszkowski M, Dauter Z, Brown JA. Structural insights into the RNA methyltransferase domain of METTL16. Sci Rep. 2018;8(1):5311. Epub 2018/03/30. doi: 10.1038/s41598-018-23608-8 . 29593291 PMC5871880

[19] Nance DJ, Satterwhite ER, Bhaskar B, Misra S, Carraway KR, Mansfield KD. Characterization of METTL16 as a cytoplasmic RNA binding protein. PLoS One. 2020;15(1):e0227647. Epub 2020/01/16. doi: 10.1371/journal.pone.0227647 . 31940410 PMC6961929

[20] Ruszkowska A. METTL16, Methyltransferase-Like Protein 16: Current Insights into Structure and Function. Int J Mol Sci. 2021;22(4). Epub 2021/03/07. doi: 10.3390/ijms22042176 . 33671635 PMC7927073

[21] Su R, Dong L, Li Y, Gao M, He PC, Liu W, et al. METTL16 exerts an m(6)A-independent function to facilitate translation and tumorigenesis. Nat Cell Biol. 2022;24(2):205–16. Epub 2022/02/12. doi: 10.1038/s41556-021-00835-2 . 35145225 PMC9070413

[22] Warda AS, Kretschmer J, Hackert P, Lenz C, Urlaub H, Höbartner C, et al. Human METTL16 is a N(6)-methyladenosine (m(6)A) methyltransferase that targets pre-mRNAs and various non-coding RNAs. EMBO Rep. 2017;18(11):2004–14. Epub 2017/10/21. doi: 10.15252/embr.201744940 . 29051200 PMC5666602

[23] Brown JA, Kinzig CG, DeGregorio SJ, Steitz JA. Methyltransferase-like protein 16 binds the 3’-terminal triple helix of MALAT1 long noncoding RNA. Proc Natl Acad Sci U S A. 2016;113(49):14013–8. Epub 2016/11/23. doi: 10.1073/pnas.1614759113 . 27872311 PMC5150381

[24] Svobodová Kovaříková A, Stixová L, Kovařík A, Komůrková D, Legartová S, Fagherazzi P, et al. N(6)-Adenosine Methylation in RNA and a Reduced m(3)G/TMG Level in Non-Coding RNAs Appear at Microirradiation-Induced DNA Lesions. Cells. 2020;9(2). Epub 2020/02/09. doi: 10.3390/cells9020360 . 32033081 PMC7072662

[25] Chen PB, Shi GX, Liu T, Li B, Jiang SD, Zheng XF, et al. Oxidative Stress Aggravates Apoptosis of Nucleus Pulposus Cells through m(6)A Modification of MAT2A Pre-mRNA by METTL16. Oxid Med Cell Longev. 2022;2022:4036274. Epub 2022/01/25. doi: 10.1155/2022/4036274 publication of this paper. 35069973 PMC8767407

[26] Sornjai W, Lithanatudom P, Erales J, Joly P, Francina A, Hacot S, et al. Hypermethylation of 28S ribosomal RNA in β-thalassemia trait carriers. Int J Biol Macromol. 2017;94(Pt A):728–34. Epub 2016/10/22. doi: 10.1016/j.ijbiomac.2016.10.039 . 27765567

[27] Ruan H, Yang F, Deng L, Yang D, Zhang X, Li X, et al. Author Correction: Human m(6)A-mRNA and lncRNA epitranscriptomic microarray reveal function of RNA methylation in hemoglobin H-constant spring disease. Sci Rep. 2021;11(1):22339. Epub 2021/11/12. doi: 10.1038/s41598-021-01793-3 . 34759301 PMC8580971

[28] Tang W, Luo HY, Albitar M, Patterson M, Eng B, Waye JS, et al. Human embryonic zeta-globin chain expression in deletional alpha-thalassemias. Blood. 1992;80(2):517–22. Epub 1992/07/15. . 1627804

[29] Jun G, Seoh JY, Jung YJ, Jeon CH, Im JS, Park JW. Factors affecting reticulocyte enrichment by density gradient ultracentrifugation. Acta Haematol. 2009;121(4):207–11. Epub 2009/05/27. doi: 10.1159/000220334 . 19468206

[30] Wei Y, Huang YH, Skopelitis DS, Iyer SV, Costa ASH, Yang Z, et al. SLC5A3-Dependent Myo-inositol Auxotrophy in Acute Myeloid Leukemia. Cancer Discov. 2022;12(2):450–67. Epub 2021/09/18. doi: 10.1158/2159-8290.CD-20-1849 . 34531253 PMC8831445

[31] Vawter MP, Hamzeh AR, Muradyan E, Civelli O, Abbott GW, Alachkar A. Association of Myoinositol Transporters with Schizophrenia and Bipolar Disorder: Evidence from Human and Animal Studies. Mol Neuropsychiatry. 2019;5(4):200–11. Epub 2019/11/27. doi: 10.1159/000501125 . 31768373 PMC6873027

[32] Zhou S, Zhang D, Guo J, Chen Z, Chen Y, Zhang J. Long non-coding RNA NORAD functions as a microRNA-204-5p sponge to repress the progression of Parkinson’s disease in vitro by increasing the solute carrier family 5 member 3 expression. IUBMB Life. 2020;72(9):2045–55. Epub 2020/07/21. doi: 10.1002/iub.2344 . 32687247

[33] Lu X, Fan Y, Li M, Chang X, Qian J. HTR2B and SLC5A3 Are Specific Markers in Age-Related Osteoarthritis and Involved in Apoptosis and Inflammation of Osteoarthritis Synovial Cells. Front Mol Biosci. 2021;8:691602. Epub 2021/07/06. doi: 10.3389/fmolb.2021.691602 . 34222340 PMC8241908

[34] De Paepe B, Merckx C, Jarošová J, Cannizzaro M, De Bleecker JL. Myo-Inositol Transporter SLC5A3 Associates with Degenerative Changes and Inflammation in Sporadic Inclusion Body Myositis. Biomolecules. 2020;10(4). Epub 2020/04/03. doi: 10.3390/biom10040521 . 32235474 PMC7226596

[35] Johnson ZI, Doolittle AC, Snuggs JW, Shapiro IM, Le Maitre CL, Risbud MV. TNF-α promotes nuclear enrichment of the transcription factor TonEBP/NFAT5 to selectively control inflammatory but not osmoregulatory responses in nucleus pulposus cells. J Biol Chem. 2017;292(42):17561–75. Epub 2017/08/27. doi: 10.1074/jbc.M117.790378 . 28842479 PMC5655530

[36] Cabrera-Cruz H, Oróstica L, Plaza-Parrochia F, Torres-Pinto I, Romero C, Vega M. The insulin-sensitizing mechanism of myo-inositol is associated with AMPK activation and GLUT-4 expression in human endometrial cells exposed to a PCOS environment. Am J Physiol Endocrinol Metab. 2020;318(2):E237–e48. Epub 2019/12/25. doi: 10.1152/ajpendo.00162.2019 . 31874063

[37] Lin L, Fang T, Lin L, Ou Q, Zhang H, Chen K, et al. Genetic Variants Relate to Fasting Plasma Glucose, 2-Hour Postprandial Glucose, Glycosylated Hemoglobin, and BMI in Prediabetes. Front Endocrinol (Lausanne). 2022;13:778069. Epub 2022/03/19. doi: 10.3389/fendo.2022.778069 . 35299963 PMC8923657

[38] Guerrieri D, Ambrosi NG, Romeo H, Salaberry J, Toniolo MF, Remolins C, et al. Secretory Leukocyte Proteinase Inhibitor Protects Acute Kidney Injury Through Immune and Non-Immune Pathways. Shock. 2021;56(6):1019–27. Epub 2021/04/22. doi: 10.1097/SHK.0000000000001785 . 33882512

[39] Cui Z, Mu C, Wu Z, Pan S, Cheng Z, Zhang ZQ, et al. The sodium/myo-inositol co-transporter SLC5A3 promotes non-small cell lung cancer cell growth. Cell Death Dis. 2022;13(6):569. Epub 2022/06/28. doi: 10.1038/s41419-022-05017-y . 35760803 PMC9237060

[40] He S, Zhang Q, Chen BY, Huang P, Tang YQ, Wei Y, et al. [Genotypes and clinical features of 595 children with HbH disease in Guangxi, China]. Zhongguo Dang Dai Er Ke Za Zhi. 2015;17(9):908–11. Epub 2015/09/29. . 26412168

[41] Chui DH, Fucharoen S, Chan V. Hemoglobin H disease: not necessarily a benign disorder. Blood. 2003;101(3):791–800. Epub 2002/10/24. doi: 10.1182/blood-2002-07-1975 . 12393486

[42] Liu Y, Wang J. Effects of DMSA-coated Fe3O4 nanoparticles on the transcription of genes related to iron and osmosis homeostasis. Toxicol Sci. 2013;131(2):521–36. Epub 2012/10/23. doi: 10.1093/toxsci/kfs300 . 23086747

[43] Bou-Fakhredin R, Dia B, Ghadieh HE, Rivella S, Cappellini MD, Eid AA, et al. CYP450 Mediates Reactive Oxygen Species Production in a Mouse Model of β-Thalassemia through an Increase in 20-HETE Activity. Int J Mol Sci. 2021;22(3). Epub 2021/01/28. doi: 10.3390/ijms22031106 . 33498614 PMC7865490

[44] Chaichompoo P, Qillah A, Sirankapracha P, Kaewchuchuen J, Rimthong P, Paiboonsukwong K, et al. Abnormal red blood cell morphological changes in thalassaemia associated with iron overload and oxidative stress. J Clin Pathol. 2019;72(8):520–4. Epub 2019/04/24. doi: 10.1136/jclinpath-2019-205775 . 31010830

[45] Sun R, Tian X, Li Y, Zhao Y, Wang Z, Hu Y, et al. The m6A reader YTHDF3-mediated PRDX3 translation alleviates liver fibrosis. Redox Biol. 2022;54:102378. Epub 2022/07/03. doi: 10.1016/j.redox.2022.102378 . 35779442 PMC9287738

[46] Liu D, Li Z, Zhang K, Lu D, Zhou D, Meng Y. N(6)-methyladenosine reader YTHDF3 contributes to the aerobic glycolysis of osteosarcoma through stabilizing PGK1 stability. J Cancer Res Clin Oncol. 2022. Epub 2022/09/29. doi: 10.1007/s00432-022-04337-y . 36171455 PMC11797469

[47] Lin Y, Jin X, Nie Q, Chen M, Guo W, Chen L, et al. YTHDF3 facilitates triple-negative breast cancer progression and metastasis by stabilizing ZEB1 mRNA in an m(6)A-dependent manner. Ann Transl Med. 2022;10(2):83. Epub 2022/03/15. doi: 10.21037/atm-21-6857 . 35282088 PMC8848410

[48] Li A, Chen YS, Ping XL, Yang X, Xiao W, Yang Y, et al. Cytoplasmic m(6)A reader YTHDF3 promotes mRNA translation. Cell Res. 2017;27(3):444–7. Epub 2017/01/21. doi: 10.1038/cr.2017.10 . 28106076 PMC5339832

[49] Shi H, Wang X, Lu Z, Zhao BS, Ma H, Hsu PJ, et al. YTHDF3 facilitates translation and decay of N(6)-methyladenosine-modified RNA. Cell Res. 2017;27(3):315–28. Epub 2017/01/21. doi: 10.1038/cr.2017.15 . 28106072 PMC5339834

[50] Evan GI, Brown L, Whyte M, Harrington E. Apoptosis and the cell cycle. Curr Opin Cell Biol. 1995;7(6):825–34. Epub 1995/12/01. doi: 10.1016/0955-0674(95)80066-2 . 8608013

[51] Guo J, Zhao W, Hao W, Ren G, Lu J, Chen X. Cucurbitacin B induces DNA damage, G2/M phase arrest, and apoptosis mediated by reactive oxygen species (ROS) in leukemia K562 cells. Anticancer Agents Med Chem. 2014;14(8):1146–53. Epub 2014/06/05. doi: 10.2174/1871520614666140601220915 . 24893803

[52] Huang H, Weng H, Sun W, Qin X, Shi H, Wu H, et al. Recognition of RNA N(6)-methyladenosine by IGF2BP proteins enhances mRNA stability and translation. Nat Cell Biol. 2018;20(3):285–95. Epub 2018/02/25. doi: 10.1038/s41556-018-0045-z . 29476152 PMC5826585

[53] Elagib KE, Lu CH, Mosoyan G, Khalil S, Zasadzińska E, Foltz DR, et al. Neonatal expression of RNA-binding protein IGF2BP3 regulates the human fetal-adult megakaryocyte transition. J Clin Invest. 2017;127(6):2365–77. Epub 2017/05/10. doi: 10.1172/JCI88936 exists. 28481226 PMC5451240

[54] Tangprasittipap A, Kaewprommal P, Sripichai O, Sathirapongsasuti N, Satirapod C, Shaw PJ, et al. Comparison of gene expression profiles between human erythroid cells derived from fetal liver and adult peripheral blood. PeerJ. 2018;6:e5527. Epub 2018/09/07. doi: 10.7717/peerj.5527 . 30186694 PMC6120446

[55] de Vasconcellos JF, Tumburu L, Byrnes C, Lee YT, Xu PC, Li M, et al. IGF2BP1 overexpression causes fetal-like hemoglobin expression patterns in cultured human adult erythroblasts. Proc Natl Acad Sci U S A. 2017;114(28):E5664–e72. Epub 2017/06/28. doi: 10.1073/pnas.1609552114 . 28652347 PMC5514697

[56] Srisupundit K, Piyamongkol W, Tongsong T. Comparison of red blood cell hematology among normal, alpha-thalassemia-1 trait, and hemoglobin Bart’s fetuses at mid-pregnancy. Am J Hematol. 2008;83(12):908–10. Epub 2008/10/22. doi: 10.1002/ajh.21287 . 18932192

