# Supplementary material for: METTL16 participates in haemoglobin H disease through m6A modification
Source: PLoS One. 2024 Aug 1;19(8):e0306043. doi: 10.1371/journal.pone.0306043 (PMC11293636; doi:10.1371/journal.pone.0306043)
Supplement: S1 File — (DOCX) [file pone.0306043.s001.docx]

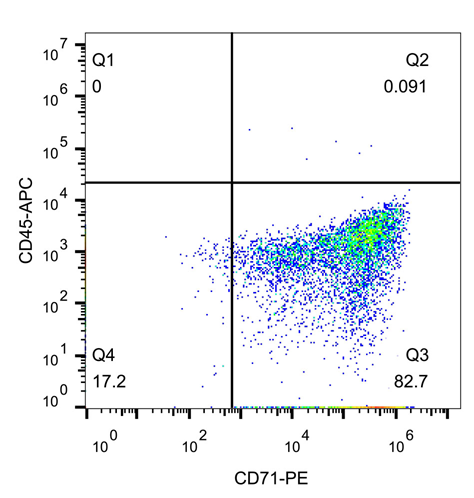


Figure S1. Confirmation of nucleated red blood cells and reticulocytes sorted through flow cytometry. Aggregation of signals in the Q3 region is indicative of a mostly (82.7%) CD71-positive and CD45-negative cell population.


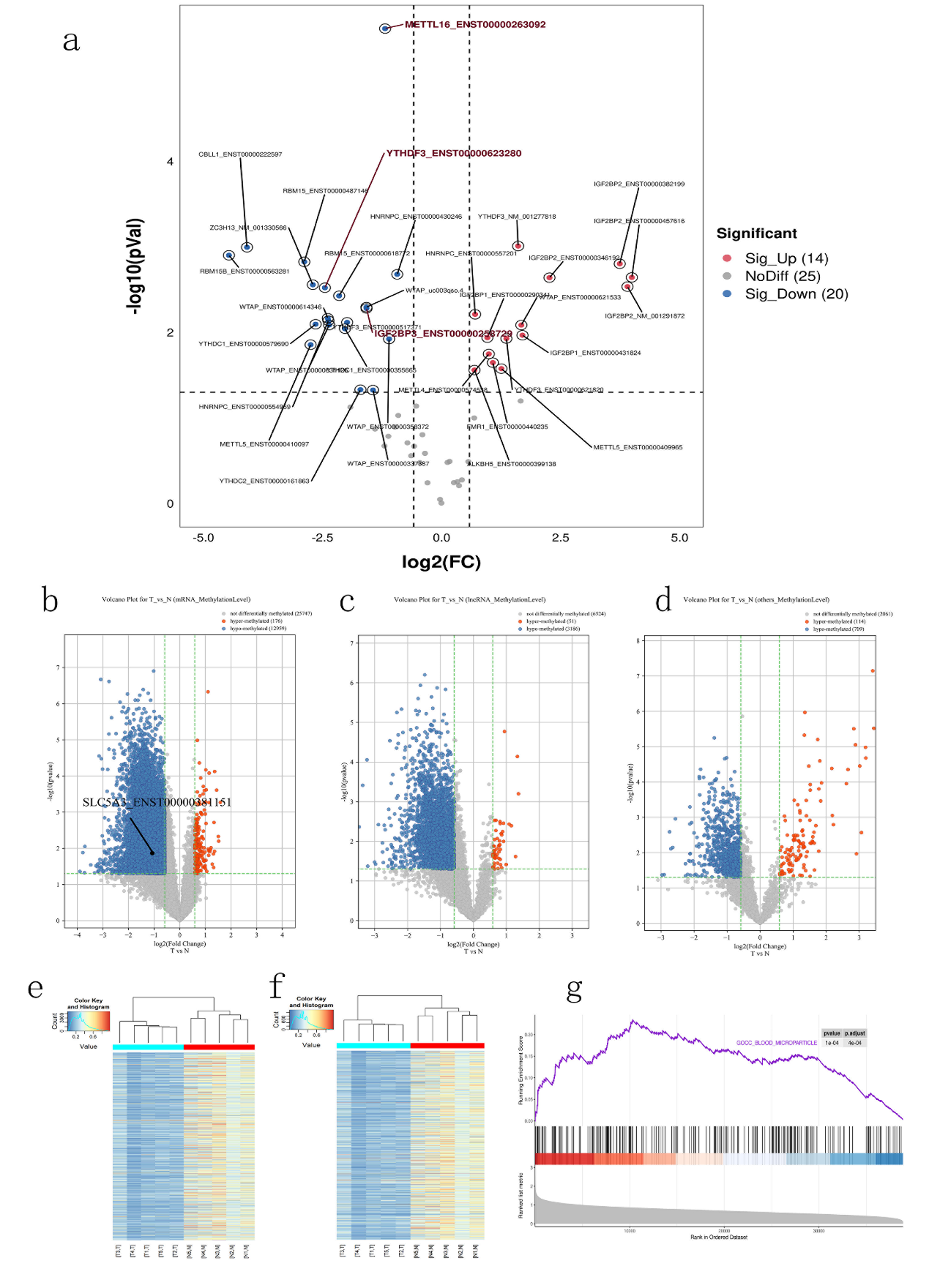


Figure S2. Differentially expressed enzymes and differential m^6^A-methylated mRNAs, lncRNAs and other small RNAs

Volcano plots showing the differentially expressed enzymes associated with methylation in the microarray (a). Volcano plots displaying differentially m^6^A-methylated mRNAs (b), lncRNAs (c) and other small RNAs (d). Heatmaps visualizing differentially methylated mRNAs (e) and lncRNAs (f). According to the results of gene set enrichment analysis (the GOCC_ BLOOD_ MICROPARTICLE was significant at *p* = 0.0001 (g).


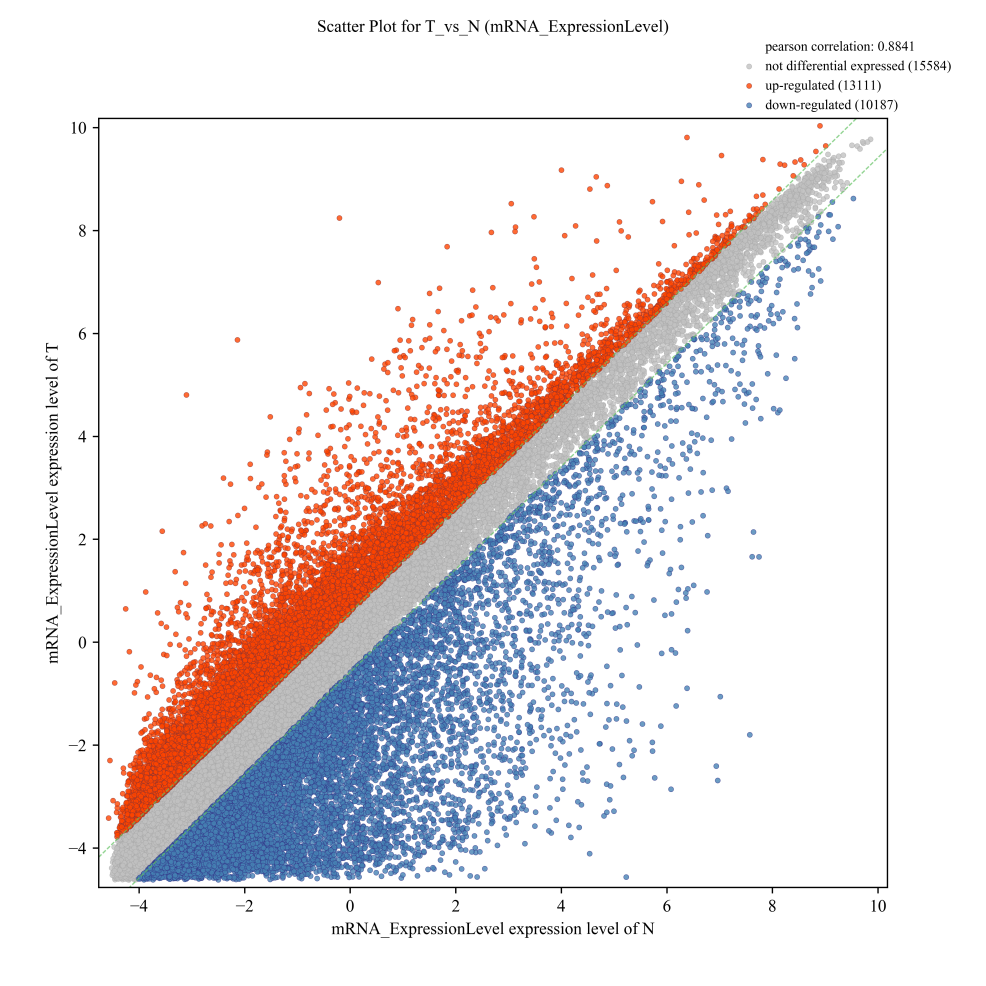


Figure S3. Scatter plots were used to visualise the differential expressions of mRNAs.





Figure S4. Heatmaps showing the differential expressions of mRNAs (a). Heatmaps were used to visualise other differentially methylated small RNAs (b). PCA diagram of mRNA expression level (c). PCA diagram of mRNA methylation level (d).





Figure S5. GO annotation of upregulated (a) and downregulated (b) mRNAs via the top 10 enrichment score-covering domains of biological processes, cellular components and molecular functions.





Figure S6. Ten significant pathways of upregulated (a) and downregulated (b) mRNAs based on enrichment scores.


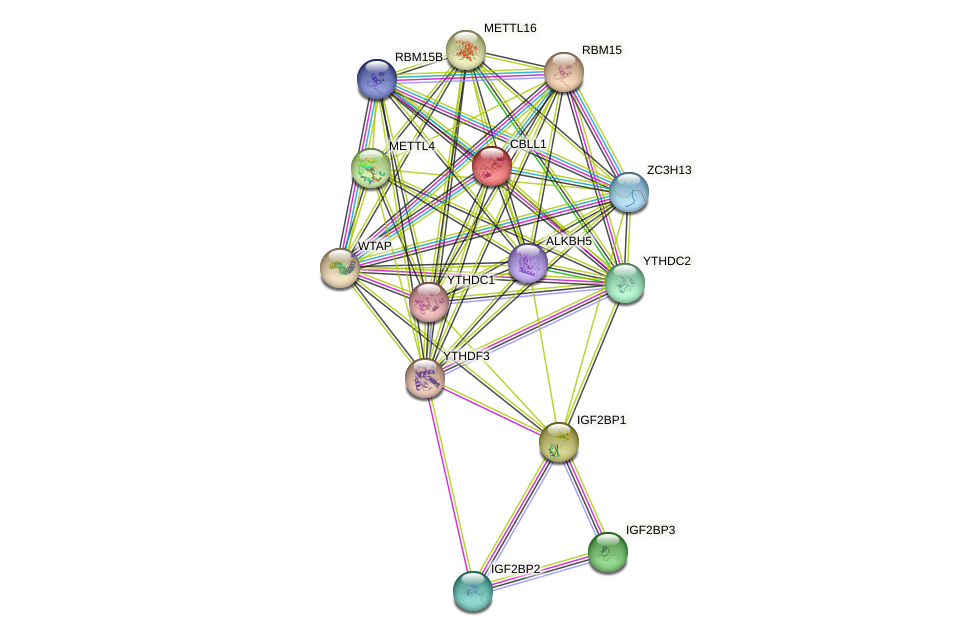


Figure S7. PPI network of differentially expressed major m^6^A-related enzymes.





Figure S8. Identification of haematological parameters related to mRNAs in HbH-CS T patients. Heatmaps showing GO and Kyoto Encyclopedia of Genes and Genomes analyses of HGB and SF-correlated m^6^A-mRNA epitranscriptomic microarray expression level (a) and methylation level (b). Heatmap showing the correlation between m^6^A-mRNA epitranscriptomic microarray expression level (c), methylation level(d) and haematological parameters HbH, HbF, HbA2, HGB, MCV, MCH, MCHC and SF in T. ＊*P*<0.05, ＊＊*P*<0.01, ＊＊＊*P*<0.001.


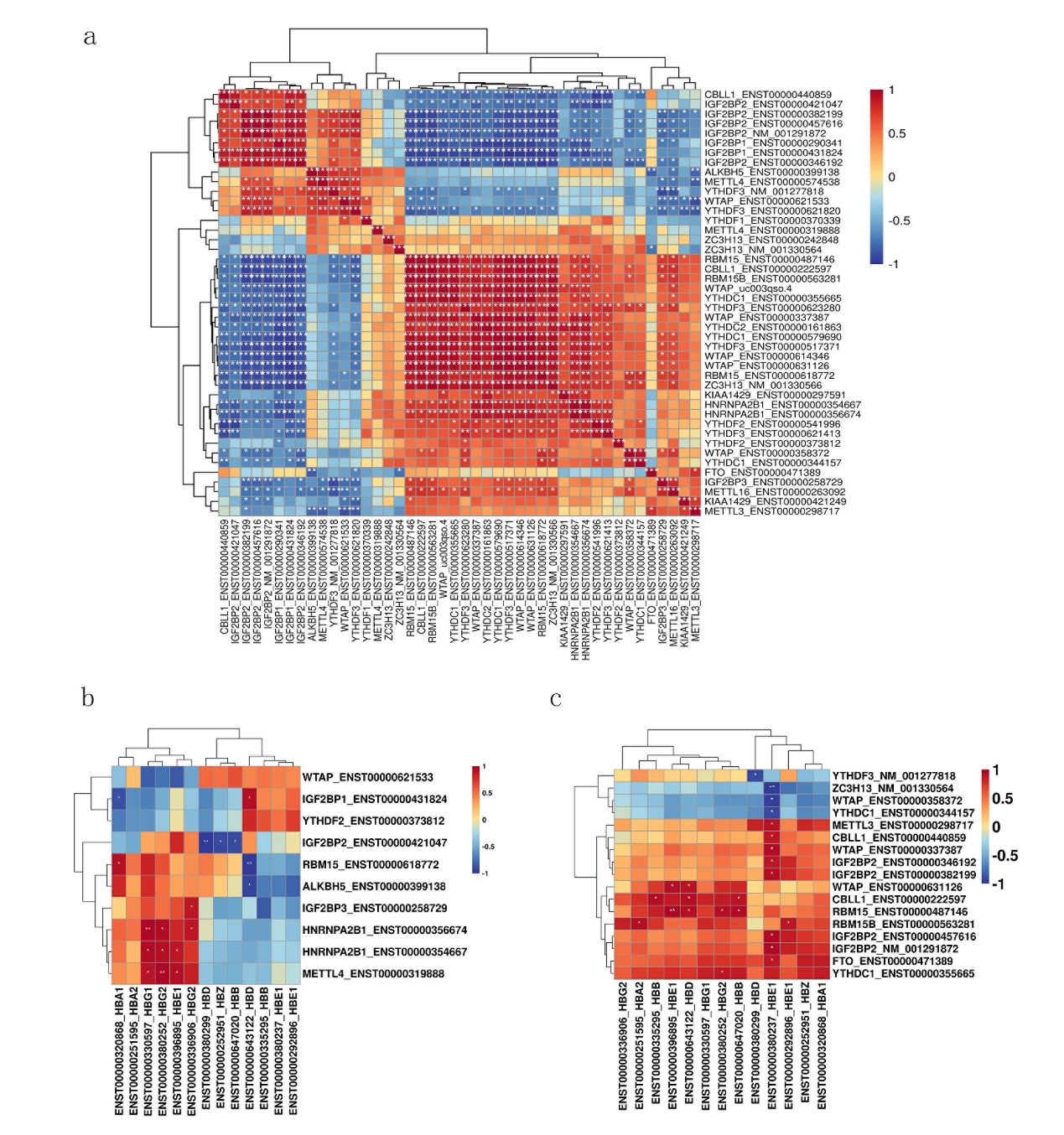


Figure S9. Heatmap showing the correlation analysis of m^6^A-related enzymes in microarray (a). Heatmap showing the correlation analysis between the expressions of various globins and those of m^6^A-related enzymes in T (b). Heatmap showing the correlation analysis between the methylation level of various globins and the expressions of m^6^A-related enzymes in T (c). ＊*P*<0.05, ＊＊*P*<0.01 and ＊＊＊*P*<0.001.





Figure S10. After 14 days of hemin treatment, no significant difference was observed in the cell cycle between K562 cells overexpressing METTL16 and the empty vector.

.


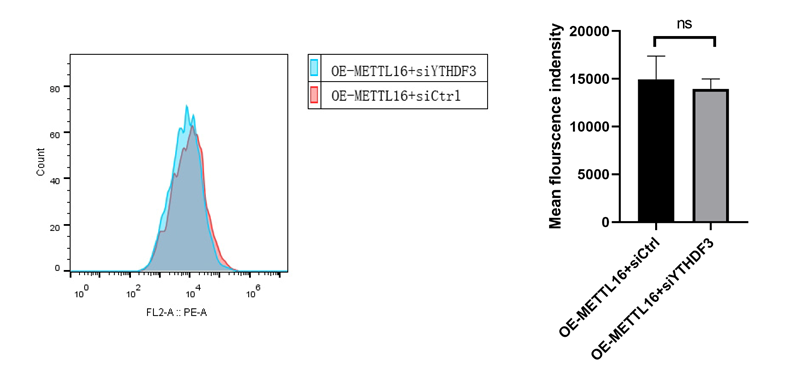


Figure S11. Effect of simultaneous knockdown of YTHDF3 on ROS in K562 cells overexpressing METTL16 (mean with SD).


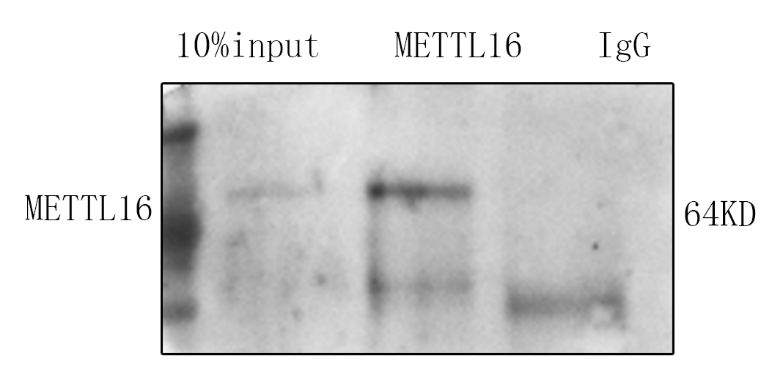


Figure S12. Western blot detection of METTL16 in METTL16-RIP protein.

Supplementary Table S1: The sequences of the qRT-PCR primers

| Primer name | Sequence (5′-3′) | | Product length (bp) |
| --- | --- | --- | --- |
| Human β-actin | forward | GTGGCCGAGGACTTTGATTG | 73 |
|  | reverse | CCTGTAACAACGCATCTCATATT |  |
| METTL16 | forward | AGTACCATCACCACCAAGTAAG | 161 |
|  | reverse | TTTCAATCCATGTCGTGACAAC |  |
| YTHDF3 | forward | GCTCCACCAACCCAACCAGTTC | 144 |
|  | reverse | CTGAGGTCCTTGTTGCTGCTGTG |  |
| IGF2BP3 | forward | GAGGCGCTTTCAGGTAAAATAG | 112 |
|  | reverse | AATGAGGCGGGATATTTCGTAT |  |
| SLC5A3 | forward | CCAGAATGTGACCAACCTGATA | 118 |
|  | reverse | TGAGAAGGCTCACAATTACAGT |  |
| HBG | forward | GGCAAGGTGAATGTGGAA | 119 |
|  | reverse | ATGATGGCAGAGGCAGAG |  |
| METTL3 | forward | AACTGCAACGCATCATTCGG | 81 |
|  | reverse | TGGTGGAACGAACCAAGCAG |  |

Supplementary Table S2: Sequences of two siRNAs

| Name | Sequence ( 5' → 3' ) |
| --- | --- |
| human-YTHDF3-siRNA-1-F | GGACGUGUGUUUAUAAUUATT |
| human-YTHDF3-siRNA-1-R | UAAUUAUAAACACACGUCCTT |
| human-YTHDF3-siRNA-2-F | CAGGCUUCAACCAGAACAATT |
| human-YTHDF3-siRNA-2-R | UUGUUCUGGUUGAAGCCUGTT |

Supplementary Table S3 Top 25 of the differentially highly expressed mRNAs

| **Gene Symbol** | **Transcript_ID** | **Fold change** | **P-value(unpaired t-test)** | **FDR** | **Locus** |
| --- | --- | --- | --- | --- | --- |
| IFIT1B | ENST00000371809 | 347.9629593 | 0.000988692 | 0.016751875 | chr10:91137813-91144962:+ |
| HBG1 | ENST00000330597 | 257.3250003 | 0.000492225 | 0.012656338 | chr11:5269309-5271089:- |
| PAQR9 | ENST00000340634 | 239.6083961 | 3.67639E-05 | 0.006378024 | chr3:142680073-142682524:- |
| ALAS2 | ENST00000335854 | 87.6710099 | 0.005282258 | 0.034189567 | chrX:55035522-55057410:- |
| CA1 | ENST00000523953 | 59.32958835 | 0.003405033 | 0.027639766 | chr8:86239837-86291243:- |
| CA1 | ENST00000524324 | 59.20789442 | 0.004596769 | 0.032009202 | chr8:86240794-86290342:- |
| KRT1 | ENST00000252244 | 58.88388949 | 0.001236572 | 0.018323319 | chr12:53068520-53074191:- |
| FECH | ENST00000262093 | 57.55710411 | 0.000206268 | 0.010264906 | chr18:55215515-55254004:- |
| KIF26A | ENST00000315264 | 52.50246048 | 0.000302344 | 0.011087756 | chr14:104605377-104647231:+ |
| TBCEL | ENST00000422003 | 48.65602794 | 6.4615E-05 | 0.007303191 | chr11:120894830-120961484:+ |
| MARCH3 | ENST00000308660 | 48.06154501 | 7.32055E-06 | 0.004508777 | chr5:126203406-126366500:- |
| C20orf100 | ENST00000341197 | 47.61581786 | 0.025385746 | 0.076962851 | chr20:42543504-42697657:+ |
| ADAT1 | ENST00000307921 | 44.24400871 | 0.040476781 | 0.102379212 | chr16:75633900-75657198:- |
| E2F2 | ENST00000361729 | 40.77480472 | 0.001508466 | 0.019794868 | chr1:23832922-23857712:- |
| SFRP2 | ENST00000274063 | 40.39759403 | 0.00116893 | 0.017872722 | chr4:154701744-154710272:- |
| STXBP6 | NM_001351943 | 40.0607569 | 0.026489251 | 0.078760807 | chr14:25278861-25519095:- |
| GUCA2B | ENST00000372581 | 39.01497142 | 0.032685907 | 0.089531062 | chr1:42619092-42621495:+ |
| HBE1 | ENST00000396895 | 38.62591998 | 0.020727448 | 0.068484418 | chr11:5289582-5291175:- |
| ITLN1 | ENST00000326245 | 38.32514794 | 0.00025656 | 0.010661864 | chr1:160846329-160854960:- |
| BPGM | ENST00000393132 | 37.87207309 | 0.004021617 | 0.029864113 | chr7:134331583-134364565:+ |
| SLC14A1 | ENST00000321925 | 37.05249136 | 0.001880617 | 0.021495312 | chr18:43304092-43332485:+ |
| ZNF248 | ENST00000374648 | 36.30808449 | 0.018130805 | 0.063490812 | chr10:38119784-38146486:- |
| HBG2 | ENST00000380252 | 35.95585913 | 0.005607906 | 0.035146173 | chr11:5274420-5526835:- |
| FBXL13 | ENST00000313221 | 35.03968066 | 0.016676596 | 0.060534113 | chr7:102453680-102715015:- |
| PTH2R | ENST00000617735 | 34.46755513 | 0.016129654 | 0.059271637 | chr2:209224438-209358754:+ |
