# Supplementary material for: METTL16 participates in haemoglobin H disease through m6A modification
Source: PLoS One. 2024 Aug 1;19(8):e0306043. doi: 10.1371/journal.pone.0306043 (PMC11293636; doi:10.1371/journal.pone.0306043)
Supplement: S1 Raw images — (PDF) [file pone.0306043.s010.pdf]

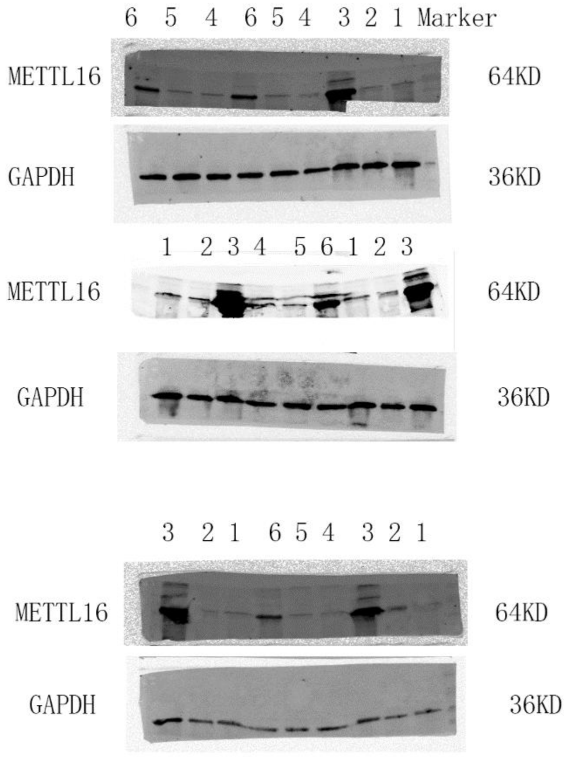

Figure 1． Fluorescent imaging. 1. Blank, 2. OE-NC, 3. OE-METTL16, 4. Hemin, 5. OE-NC+Hemin, and 6. OE-METTL16+Hemin

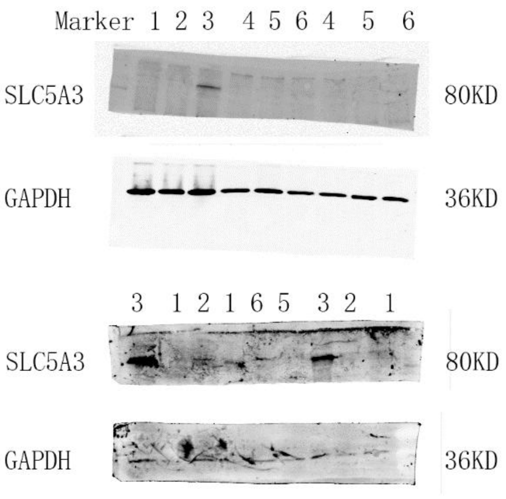

Figure 2 . Fluorescent imaging. 1. Blank, 2. OE-NC, 3. OE-METTL16, 4. Hemin, 5. OE-NC+Hemin, and 6. OE-METTL16+Hemin

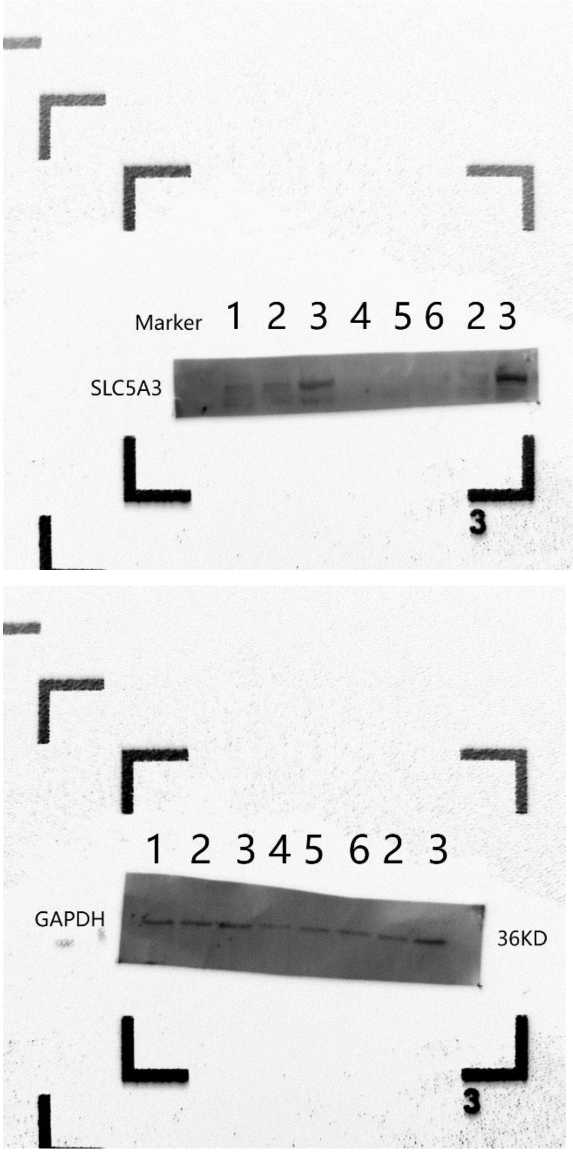

Figure 3． Chemiluminescence imaging. 1. Blank, 2. OE-NC, 3. OE-METTL16, 4. Hemin, 5. OE-NC+Hemin, and 6. OE-METTL16+Hemin

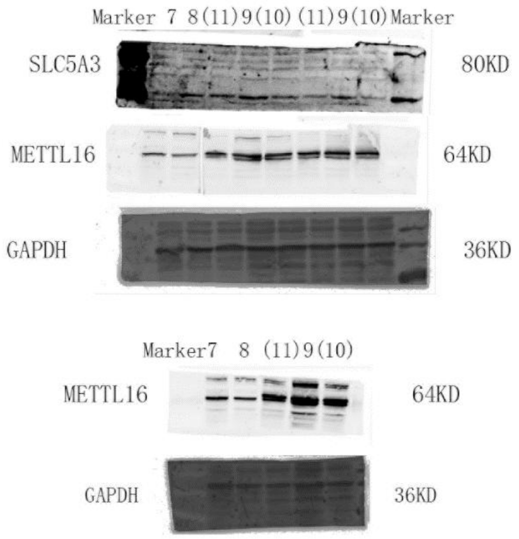

Figure 4． Fluorescent imaging. 7. shCtrl, 8. shMETTL16, 9. OE-METTL16+siCtrl, (10). OE-METTL16+siYTHDF3-1, and (11). OE-METTL16+siYTHDF3-2.

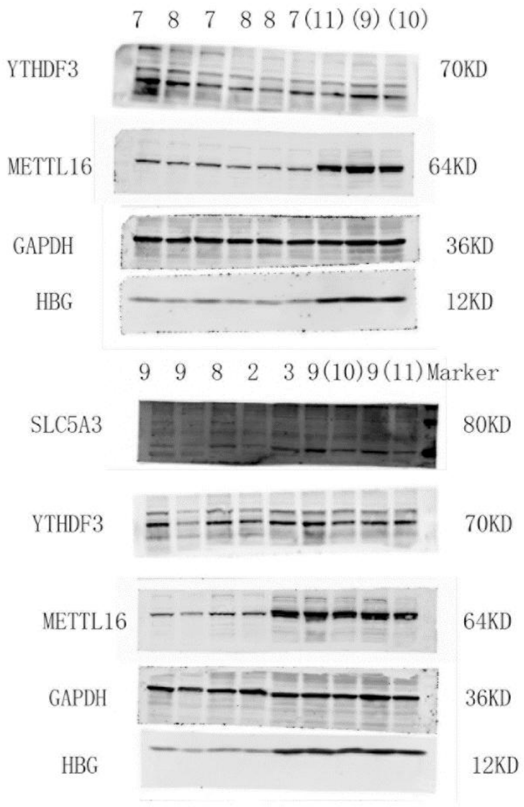

Figure 5. Using chemiluminescence imaging to detect YTHDF3, using fluorescence imaging to detect SLC5A3, METTL16, GAPDH, and HBG. 7. shCtrl, 8. shMETTL16, 9. OE-METTL16+siCtrl, (10). OE-METTL16+siYTHDF3-1, and (11). OE-METTL16+siYTHDF3-2.

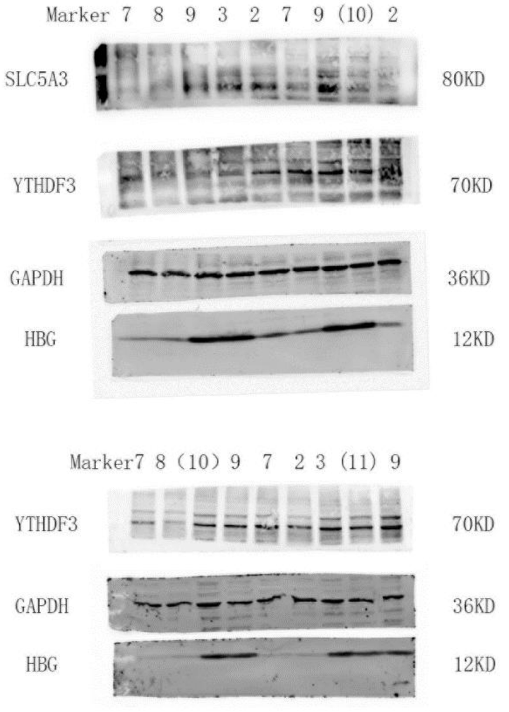

Figure 6．Using chemiluminescence imaging to detect YTHDF3 and SLC5A3, using fluorescence imaging to detect GAPDH and HBG. 7. shCtrl, 8. shMETTL16, 9. OE-METTL16+siCtrl, (10). OE-METTL16+siYTHDF3-1, and (11). OE-METTL16+siYTHDF3-2.

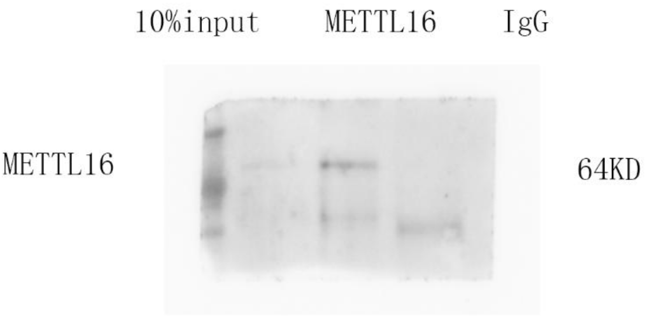

Figure 7．Chemiluminescence imaging.
